# A General Framework for Neutrality Tests Based on the Site Frequency Spectrum

**DOI:** 10.3390/genes14091714

**Published:** 2023-08-28

**Authors:** Sebastián E. Ramos-Onsins, Giacomo Marmorini, Guillaume Achaz, Luca Ferretti

**Affiliations:** 1 Centre for Research in Agricultural Genomics (CRAG) CSIC-IRTA-UAB-UB, 08193 Bellaterra, Spain; 2 Department of Physics and Mathematics, Aoyama Gakuin University, Sagamihara 252-5258, Japan; giacomo@yukawa.kyoto-u.ac.jp; 3 Department of Physics, Nihon University, Tokyo 156-8550, Japan; 4 Institut de Systématique, Evolution, Biodiversité, UMR 7205, MNHN and Centre Interdisciplinaire de Recherche en Biologie, UMR 7241, Collége de France, 75231 Paris, France; guillaume.achaz@mnhn.fr; 5 Pandemic Sciences Institute and Big Data Institute, Nuffield Department of Medicine, University of Oxford, Oxford OX1 3AZ, UK

**Keywords:** coalescent theory, site frequency spectrum, neutrality test, statistical power, summary statistics

## Abstract

One of the main necessities for population geneticists is the availability of sensitive statistical tools that enable to accept or reject the standard Wright–Fisher model of neutral evolution. A number of statistical tests have been developed to detect specific deviations from the null frequency spectrum in different directions (e.g., Tajima’s D, Fu and Li’s F and D tests, Fay and Wu’s H). A general framework exists to generate all neutrality tests that are linear functions of the frequency spectrum. In this framework, it is possible to develop a family of optimal tests with almost maximum power against a specific alternative evolutionary scenario. In this paper we provide a thorough discussion of the structure and properties of linear and nonlinear neutrality tests. First, we present the general framework for linear tests and emphasise the importance of the property of scalability with the sample size (that is, the interpretation of the tests should not depend on the sample size), which, if missing, can lead to errors in interpreting the data. After summarising the motivation and structure of linear optimal tests, we present a more general framework for the optimisation of linear tests, leading to a new family of tunable neutrality tests. In a further generalisation, we extend the framework to nonlinear neutrality tests and we derive nonlinear optimal tests for polynomials of any degree in the frequency spectrum.

## 1. Introduction

Since the development of molecular genetics techniques allowed to obtain nucleotide sequences for the study of populations genetics [1], a number of neutrality tests have been developed with the objective to facilitate the interpretation of an increasing volume of molecular data. Statistical tests for neutrality have been generated exploiting the different properties of the stationary neutral model. Examples of tests are the HKA [2], which takes advantage of the polymorphism/divergence relationship across independent loci in a multilocus framework, and the Lewontin–Krakauer test [3], which looks for an unexpected level of population differentiation in a locus in relation to other loci. Additionally, another family of popular tests are the ones related to linkage disequilibrium, such as the one developed by [4], which detects long haplotypes at unusual elevated frequencies in candidate regions.

An important family of these tests, often used as summary statistics, is built on the frequency spectrum of biallelic polymorphisms. This family includes the well known tests by Tajima [5], Fu and Li [6], and Fay and Wu [7]. If an outgroup is available, these tests are based on the unfolded spectrum $\xi_{i}$, that is, the number of segregating sites with a derived allele frequency of *i* in a sample of (haploid) size *n*. Without an outgroup, it is not possible to distinguish derived and ancestral alleles and the only available data correspond to the folded spectrum $\eta_{i}$, that is, the number of segregating sites with a minor allele frequency of *i*. The quantities $\xi_{i}$ and $\eta_{i}$ are all positive and the range of allele frequencies is 1≤i≤n−1 for the unfolded spectrum, 1≤i≤⌊n/2⌋ for the folded spectrum. The average spectra for the standard Wright–Fisher neutral model are given by:

(1) $$E\left(\xi_{i}\right)=\frac{1}{i}\theta L\;,\;E\left(\eta_{i}\right)=\frac{n}{i\left(n-i\right)\left(1+\delta_{i,n-i}\right)}\theta L\;,$$

where *L* is the length of the sequence and $\theta =2p\mu N_{e}$, where μ is the mutation rate per base, *p* is the ploidy and $N_{e}$ is the effective population size. Note that we define θ as the rescaled mutation rate per base and not per sequence. Apart from this, we follow the notation of [8,9]. Note that the spectra are proportional to θ.

A general framework for linear tests was presented in [8]. The general tests proposed there were of the form:

(2) $$T_{\Omega }=\frac{\sum_{i=1}^{n-1}i\Omega_{i}\xi_{i}}{\sqrt{\mathrm{Var}\left(\sum_{j=1}^{n-1}j\Omega_{j}\xi_{j}\right)}}\;,\;T_{\Omega }^{*}=\frac{\sum_{i=1}^{\left\lfloor n/2\right\rfloor }i\Omega_{i}^{*}\eta_{i}}{\sqrt{\mathrm{Var}\left(\sum_{j=1}^{\left\lfloor n/2\right\rfloor }j\Omega_{j}^{*}\eta_{j}\right)}}$$

that are centred (i.e., they have a null expectation value) if the weights $\Omega_{i},\Omega_{i}^{*}$ satisfy the conditions $\sum_{i=1}^{n-1}\Omega_{i}=0$ and $\sum_{i=1}^{\left\lfloor n/2\right\rfloor }\Omega_{i}^{*}=0$. This framework allows the construction of many new neutrality tests and has been used to obtain optimal tests for specific alternative scenarios [10]. However, the original framework does not take into account the dependence of the tests on the sample size, as emphasised in [10]. It is important to choose this dependence in order to obtain results that are as independent as possible on sample size. Moreover, the framework presented in [8] covers just a large subfamily of neutrality tests based on the frequency spectrum, that is, the class of tests that are linear functions of the spectrum. This subfamily contains almost all the tests that can be found in the literature with the exception of the $G_{\xi },G_{\eta }$ tests of Fu [11], which are second order polynomials in the spectrum whose form is related with Hotelling statistics. Since these $G_{\xi },G_{\eta }$ tests were shown to be quite effective in some scenarios, it would be interesting to build a general framework for these quadratic (and more generally nonlinear) tests. New optimal tests based on extensions of the site frequency spectrum [12,13] were recently applied successfully to the detection of selection pressure on human chromosomal inversions [14].

In this work we provide a detailed study of the properties of the whole family of tests based on the site frequency spectrum, i.e., focus on the structure and the properties of neutrality tests that consider the frequency of the variants per position. Technical details and proofs can be found in Appendix A and Appendix B.

We begin with the discussion of the most interesting case, i.e., linear tests. Achaz [8] developed a general framework for constructing linear tests comparing two different estimators of variability, which are based on linear combinations of the frequency, each one containing different weights. We summarise his approach in the Section 2.1.

In Section 2.2, we discuss the importance of scaling the weights with the aim to avoid a dependence with the sample size. This allows us to interpret the information of the test in the same way for any sample size analysed. We provide a thorough analysis of a simple proposal for the scaling of the tests with the sample size. Different scaling strategies (including alternatives to scaling) are analysed and evaluated, demonstrating the importance but also the suitability of different weighting methods depending on the nature of the statistic.

In Section 2.3, we present and expand the theory of optimal tests, i.e., tests that have maximum power to detect an alternative scenario versus a null scenario, introduced in [10]. We show that generic linear tests cannot detect arbitrary deviations from the neutral spectrum, and why tests must be optimised with respect to a specific alternative scenario. A geometrical interpretation of neutrality test is developed, showing how these tests depend on the differences between the null and the alternative scenario, clarifying the theoretical basis for these tests.

In Section 2.4, we extend this approach to nonlinear tests that consider different moments of the frequency spectrum combined in generic polynomials or in power series. In contrast to linear tests, nonlinear tests are dependent on the level of variability θ and thus need an accurate estimation of θ to have good statistical properties (e.g., unbiased, high statistical power). These tests are classified in strongly and weakly centred (that is, having null expectation value). Strongly centred tests are those tests that are centred for any value for any estimate of θ, even if this estimate is far from the actual value, and thus are more robust to deviations of θ estimates than weakly centred tests (simulations shown already in the Results section (Section 3)). Instead, weakly centred tests are only centred if the θ estimate is equal to the actual value, but this feature also makes the tests more powerful if the inference of θ is reliable.

In Section 3.1 in the Results, we see some consequences of the framework outlined in Methods; it is possible to optimise neutrality tests following a general maximisation approach that depends on the proxy used for the power to reject the null model in the alternative scenario, which depends on the mean spectra, the covariance matrices, and the critical *p*-value used. Moreover, we show that under some conditions, the power maximisation can be always achieved by tuning a parameter in a new family of linear “tunable” optimal tests developed here, which depend only on the mean spectra of the alternative model.

In Section 3.2, we present the results for the power to detect deviations from the neutral spectrum in coalescent simulations. We show how linear optimal tests have more power than usual neutrality tests such as Tajima’s *D*, nonlinear optimal tests are more powerful than linear ones, and weakly centred tests are more powerful than strongly centred ones if θ is known.

In summary, our research augments the understanding of neutrality tests, encompassing weight normalisation, optimal test formulation, and both linear and nonlinear paradigms. The outcome not only enriches theoretical foundations but also provides novel methodologies for increasing power and effectiveness of neutrality tests across diverse scenarios.

## 2. Material and Methods

### 2.1. Linear Neutrality Tests

#### General Framework

As discussed by Achaz [8], the general form for linear tests based on the unfolded spectrum can be written as:

(3) $$T_{\Omega }=\frac{\sum_{i=1}^{n-1}i\Omega_{i}\xi_{i}}{\sqrt{\mathrm{Var}\left(\sum_{j=1}^{n-1}j\Omega_{j}\xi_{j}\right)}}$$

where $\Omega_{i}$ is a set of weights satisfying the condition:

(4) $$\sum_{i=1}^{n-1}\Omega_{i}=0\;.$$

This is the most general form if we require that the test is centred and with variance 1, that is, $E(T_{\Omega })=0$ and $\mathrm{Var}(T_{\Omega })=1$. The condition of intendedness can be obtained substituting the spectrum with its average in the standard neutral model, given by the Equation (1).

Alternatively, it is sufficient to choose any pair of unbiased estimators of θ based on the unfolded spectrum:

(5) $${\hat{\theta }}_{\omega }=\frac{1}{L}\sum_{i=1}^{n-1}i\omega_{i}\xi_{i}\;,\;{\hat{\theta }}_{\omega^{\prime }}=\frac{1}{L}\sum_{i=1}^{n-1}i\omega_{i}^{\prime }\xi_{i}$$

with weights $\omega_{i},\omega_{i}^{\prime }$ that obey the conditions:

(6) $$\sum_{i=1}^{n-1}\omega_{i}=1\;,\;\sum_{i=1}^{n-1}\omega_{i}^{\prime }=1$$

to obtain a new test for neutrality:

(7) $$T_{\Omega }=\frac{{\hat{\theta }}_{\omega }-{\hat{\theta }}_{\omega^{\prime }}}{\sqrt{\mathrm{Var}({\hat{\theta }}_{\omega }-{\hat{\theta }}_{\omega^{\prime }})}}=\frac{\sum_{i=1}^{n-1}i\left(\omega_{i}-\omega_{i}^{\prime }\right)\xi_{i}}{\sqrt{\mathrm{Var}\left(\sum_{j=1}^{n-1}j\left(\omega_{j}-\omega_{j}^{\prime }\right)\xi_{j}\right)}}=\frac{\sum_{i=1}^{n-1}i\Omega_{i}\xi_{i}}{\sqrt{\mathrm{Var}\left(\sum_{j=1}^{n-1}j\Omega_{j}\xi_{j}\right)}}$$

that is equivalent to the definition (3) with $\Omega_{i}=\omega_{i}-\omega_{i}^{\prime }$. Therefore a test $T_{\Omega }$ is defined by real vectors Ω or $\omega ,\omega^{\prime }$ satisfying the above normalisation conditions.

### 2.2. Sample Size Independent Tests

#### 2.2.1. Scaling of Weights with Sample Size

In this section we would like to remark that there are conditions that have to be imposed on the weights $\Omega_{i}$ or $\omega_{i},\omega_{i}^{\prime }$ to ensure that these tests are consistent and meaningful in their interpretation. In fact, the values (and even the number!) of these weights depend explicitly on sample size *n*. Since every conceivable test should be applied to samples of different sizes, then its definition involves a whole family of weights $\left\{\Omega_{i}^{\left(n\right)}\right\}$ or $\left\{\omega_{i}^{\left(n\right)},\omega_{i}^{\prime (n)}\right\}$ with n=2,3…∞ and to define a test it is necessary to specify how these weights scale with *n*.

**Figure 1:**
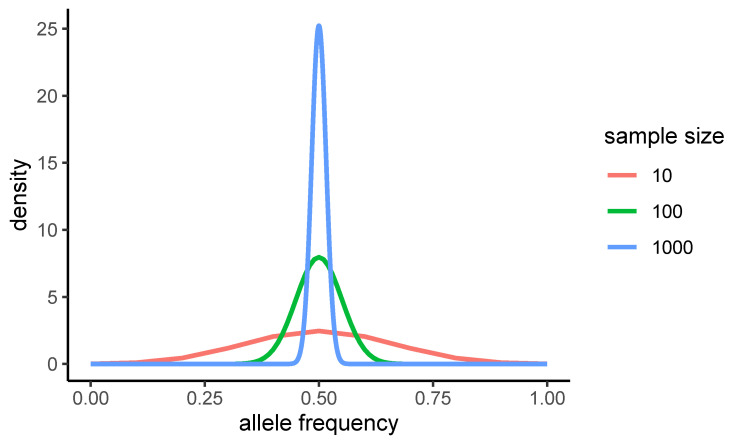
Illustrative example of the dependence of the weight on sample size: weight Ω as a function of i/n for the test for admixture by Achaz, plotted for different sample size n=10 (blue), 100 (red), 1000 (green).

As an example of the weird effects of some choices of scaling, we consider the test for admixture of [8]. The weights of this test are ωi=ni2−n(1−2−n+1)−1 and $\omega_{i}^{\prime }=1/\left(n-1\right)$. Suppose that the population under study shows an excess of alleles of frequency *f* between 0.3 and 0.4. The average weight of these frequencies, rescaled by the sample size, is 0.5 for n=10, but it reduces to −0.75 for n=100 and to −1.0 for n=1000. These weights are largely different, even in sign, therefore a strong excess of alleles in this range of frequency would show itself as either a positive or a negative value for this test, depending on the sample size! The reason can be understood by noticing that for *n* large, the binomial can be approximated by a Gaussian function of the allele frequencies f=i/n centred in f=1/2 and with variance 1/4n. Therefore this weight function has a strong dependence on *n* when considered as a function of *f* and *n*. The changes of this weight function with sample size are apparent in the plot of Figure 1, which shows the actual function (rescaled by sample size) for n= 101,001,000.

In this example it is apparent that the interpretation of the results of this test depends on *n*. This means that the calibration of the test should be different for each possible sample size.

The weight-consistency requirement that we propose is that the result of the test should be almost independent of sample size. This requirement is equivalent to a condition on the scaling of the weights $\Omega_{i}^{\left(n\right)}$ with *n*. Our proposal for a reasonable requirement on this scaling is the following: the relative weight of different frequencies in the population should remain approximately constant while varying the size of the sample. This condition ensures that at least for sufficiently large *n*, the average values of the test on samples of different size from the same population should be approximately independent on sample size, i.e., that the test should be weight-consistent. Note that this requirement has to do with the interpretation of the test, rather than with the usual definition of statistical consistency. Tests that are not weight-consistent could be statistically consistent, but the interpretation of results in the left and right tails would not be assured to be independent of sample size.

To determine the scaling, we note that in limit of large *n*, the frequency spectrum approaches a continuum and we can define the weights as functions Ω(f) or $\omega \left(f\right),\omega^{\prime }\left(f\right)$ with f∈(0,1) and $\int_{0}^{1}df\;\Omega \left(f\right)=0$, $\int_{0}^{1}df\;\omega \left(f\right)=\int_{0}^{1}df\;\omega^{\prime }\left(f\right)=1$. Since the ratio of the derived allele count and the sample size i/n is an unbiased estimator of the frequency *f* of the allele in the population (because E(i)=nf), a simple scaling that satisfies the above requirement is:

(8) $$\Omega_{i}^{\left(n\right)}≃\Omega \left(i/n\right)\;\mathrm{or}\;\omega_{i}^{\left(n\right)}≃\omega \left(i/n\right)\;,\;\omega_{i}^{\prime (n)}≃\omega^{\prime }\left(i/n\right)$$

as proposed by some of the authors in [10].

In order to have the above approximate scaling while obeying the condition $\sum_{i=1}^{n-1}\Omega_{i}=0$, there are two simple weight-consistent forms for the weights:

(9) $$\Omega_{i}^{\left(n\right)}=\Omega \left(\frac{i}{n}\right)-\frac{1}{n-1}\sum_{j=1}^{n-1}\Omega \left(\frac{j}{n}\right)$$

where the last term is a (typically small) correction that enforce centredness of the test, or:

(10) $$\Omega_{i}^{\left(n\right)}=\omega_{i}^{\left(n\right)}-\omega_{i}^{\prime (n)}=\frac{\omega \left(\frac{i}{n}\right)}{\sum_{j=1}^{n-1}\omega \left(\frac{j}{n}\right)}-\frac{\omega^{\prime }\left(\frac{i}{n}\right)}{\sum_{j=1}^{n-1}\omega^{\prime }\left(\frac{j}{n}\right)}$$

where the denominators are normalisation factors.

Typically, this second form (10) for the scaling is more consistent in practice and it is implicitly assumed for most of the existing tests. However, the above expressions give similar numerical results for most choices of the functions $\Omega \left(f\right)=\omega \left(f\right)-\omega^{\prime }\left(f\right)$. In fact, if Ω(f) is a limited and piecewise-continuous function, the difference between (9) and (10) is of order O(Ω)/n (since it is a factor coming from the discretisation of the frequencies) and it does not have a relevant impact on the results of the test. Therefore, in these cases the two scaling relations (9) and (10) are practically equivalent.

Note that all the tests involving the Watterson estimator (that corresponds to ω(f)∼1/f) have additional subtleties that are discussed in the next section.

##### Example: Fay and Wu’s *H* test

This test was proposed in [7] to look for an excess of high-frequency derived alleles as a signal of selection. It can be defined by the weight functions ω(f)=2(1−f) and $\omega^{\prime }\left(f\right)=2f$ or alternatively ω(f)=1 and $\omega^{\prime }\left(f\right)=2f$. The weights can be found following Equation (10). The resulting test is:

(11) $$T_{H}=\frac{\frac{1}{n-1}\sum_{i=1}^{n-1}i\xi_{i}-\frac{2}{n(n-1)}\sum_{i=1}^{n-1}i^{2}\xi_{i}}{\sqrt{\mathrm{Var}\left(\frac{1}{n-1}\sum_{j=1}^{n-1}j\xi_{j}-\frac{2}{n(n-1)}\sum_{j=1}^{n-1}j^{2}\xi_{j}\right)}}$$

The scaling defined in Equation (9), with weight function $\Omega \left(f\right)=\omega \left(f\right)-\omega^{\prime }\left(f\right)=1-2f$, gives precisely the same result.

##### Example: $F(r,r^{\prime })$ tests of Fu [15]

This large class of test is based on the comparison of two estimators with weights:

(12) $$\omega_{i}=\frac{i^{-r}}{\sum_{j=1}^{n-1}j^{-r}}\;,\;\omega_{i}^{\prime }=\frac{i^{-r^{\prime }}}{\sum_{j=1}^{n-1}j^{-r^{\prime }}}$$

that in the case $r,r^{\prime }<1$ correspond precisely to the scaling (10) suggested above, with weight functions $\omega \left(f\right)=\left(1-r\right)f^{-r}$ and $\omega^{\prime }\left(f\right)=\left(1-r^{\prime }\right)f^{-r^{\prime }}$. This can be easily verified by multiplying both the numerator and the denominator of $\omega_{i}$, $\omega_{i}^{\prime }$ by a factor $\left(1-r\right)/n^{-r}$, $\left(1-r^{\prime }\right)/n^{-r^{\prime }}$ respectively. The test by Fay and Wu corresponds actually to F(0,−1).

The cases with r≥1 or $r^{\prime }\geq 1$ involve weight functions with divergent integrals and will be discussed in the next section.

Note that the same weight functions with the scaling (9) would give rise to a slightly different test with weights:

(13) $$\Omega_{i}=\left(1-r\right){\left(\frac{i}{n}\right)}^{-r}-\left(1-r^{\prime }\right){\left(\frac{i}{n}\right)}^{-r^{\prime }}-\left(\frac{\left(1-r\right)\sum_{j=1}^{n-1}j^{-r}}{\left(n-1\right)n^{-r}}-\frac{\left(1-r^{\prime }\right)\sum_{j=1}^{n-1}j^{-r^{\prime }}}{\left(n-1\right)n^{-r^{\prime }}}\right)$$

that is not weight-consistent for weights of low frequency alleles, i.e., with $i/n≲n^{2/\max(r,r^{\prime })}$, and is therefore less interesting.

##### Example: Test for bottleneck of Achaz [8]

This test is another example of a test with an unwanted scaling:

(14) $$\omega_{i}=\frac{e^{-\alpha i}}{\sum_{j=1}^{n-1}e^{-\alpha j}}\;,\;\omega_{i}^{\prime }=\frac{1}{n-1}$$

The weight function for this test is $e^{-\alpha nf}\alpha n/\left(1-e^{-\alpha n}\right)-1$ that depends strongly on *n*, therefore this test is not weight-consistent in the above sense.

It is easy to build an equivalent test with the correct scaling by choosing the functions $\omega \left(f\right)=\beta e^{-\beta f}/\left(1-e^{-\beta }\right)$, $\omega^{\prime }\left(f\right)=1$. The resulting weights with the scaling (10) are:

(15) $$\omega_{i}=\frac{e^{-\beta i/n}}{\sum_{j=1}^{n-1}e^{-\beta j/n}}=\frac{1-e^{-\beta /n}}{1-e^{-\beta (1-1/n)}}e^{-\beta (i-1)/n}\;,\;\omega_{i}^{\prime }=\frac{1}{n-1}$$

as discussed before. The optimal value reported in [8] is α≃0.9 for n=30. This value corresponds to β≃27.

The test can also be implemented by choosing the scaling (9) and the weight function $\Omega \left(f\right)=\omega \left(f\right)-\omega^{\prime }\left(f\right)=\beta e^{-\beta f}/\left(1-e^{-\beta }\right)-1$. The resulting weights are:

(16) $$\begin{array}{cc} \Omega_{i}= & \frac{\beta e^{-\beta i/n}}{1-e^{-\beta }}-1-\frac{1}{n-1}\left(\frac{\beta (1-e^{-\beta (1-1/n)})}{\left(1-e^{-\beta }\right)e^{\beta /n}\left(1-e^{-\beta /n}\right)}-\left(n-1\right)\right)=\\ = & \frac{\beta (1-e^{-\beta (1-1/n)})}{\left(1-e^{-\beta }\right)e^{\beta /n}\left(1-e^{-\beta /n}\right)}\cdot \left(\frac{1-e^{-\beta /n}}{1-e^{-\beta (1-1/n)}}e^{-\beta (i-1)/n}-\frac{1}{n-1}\right) \end{array}$$

that are equivalent to the weights (15) up to an irrelevant multiplicative factor (see Theorem A1). Therefore, in this case the two choices of scaling give precisely the same result.

#### 2.2.2. Divergent Weights

As discussed above, the two choices of scaling in Equations (9) and (10) do not usually obtain sensibly different numerical results. However, there are important choices of Ω(f) for which this approximate equivalence between (9) and (10) does not hold. These critical cases correspond to functions that diverge as 1/f or faster near f=0 (or f=1). This divergence is not a real feature of the distribution, because the integral has a natural cutoff at the scale of the inverse population size $f_{min}=1/N$ (more precisely, the effective population size $1/N_{e}$, but this does not affect the discussion). However, in this case the integral $\int_{1/N}^{1}df\;\Omega \left(f\right)$ has a strong dependence on the cutoff 1/N and therefore the function Ω(f) itself should depend strongly on *N* to ensure proper normalisation.

If this dependence is contained in a multiplicative term in front of ω(f) or $\omega^{\prime }\left(f\right)$ or both, then the second term in Equation (9) is not a small correction of order 1/n as it happens with simple functions Ω(f), but rather it represents a relevant correction with a strong dependence on sample size *n* and population size *N*. The denominators in Equation (10) also show a strong dependence on *n* (that could not be avoided anyway) but not on *N*, and therefore this second scaling form should be used. The dependence on sample size is as strong as the dependence of the divergent integral from the cutoff. This can be easily understood by noticing that the sample size *n* plays the role of the cutoff in the sum over the frequencies that are present in the sample, which is the same role played by the population size *N* for the whole population; more formally, the denominator in Equation (10) can be bounded from above and from below by the divergent integral, and therefore the divergence of the denominator as n→∞ will be the same as the divergence of the integral as its inverse cutoff (that is, *N*) goes to infinity). For functions diverging as $f^{-k}$ with k≥1, the dependence on *n* goes as $n^{1-k}$ if k>1 or log(n) for k=1. This case always occurs when the test is built by comparing an estimator of θ with the Watterson estimator, which corresponds to ω(f)∼1/f and therefore has a logarithmic dependence on *n* given by the usual harmonic factor $a_{n}=\sum_{j=1}^{n-1}1/j≃\log\left(n\right)+\gamma +O\left(1/n\right)$. A well-known example of this case is Tajima’s *D* [5].

If the dependence of Ω(f) on *N* is contained in an additive term that does not depend on *f*, it is the correction in (9) that does not depend on *N* and therefore the first scaling form is more appropriate. We do not know examples of tests of this kind in the literature, even if the test by Zeng et al. [16] can be interpreted also in this way.

##### Example: Tajima’s *D* test

This is the most known test for neutrality based on the frequency spectrum. It is given by the difference between the Tajima estimator Π[17] based on the nucleotide pairwise diversity Π and the Watterson estimator $\theta_{W}$[18] based on the number *S* of segregating sites, therefore it can be defined by the weight functions ω(f)=2(1−f) for Π and $\omega^{\prime }\left(f\right)=1/f\log\left(N\right)$ for the Watterson estimator. The latter function has an integral that diverges logarithmically near f=0, and the corresponding dependence on *N* is contained in the factor 1/log(N) that multiplies $\omega^{\prime }\left(f\right)$, therefore the scaling (10) should be used. The result is the usual test:

(17) $$T_{D}=\frac{\sum_{i=1}^{n-1}\frac{2i(n-i)}{n(n-1)}\xi_{i}-S/a_{n}}{\sqrt{\mathrm{Var}\left(\sum_{j=1}^{n-1}\frac{2j(n-j)}{n(n-1)}\xi_{j}-S/a_{n}\right)}}=\frac{\Pi -S/a_{n}}{\sqrt{\mathrm{Var}\left(\Pi -S/a_{n}\right)}}$$

##### Example: Test of Zeng et al. [16]

This test was proposed to look for an excess of high-frequency derived alleles compared to low-frequency alleles. It is defined by the weight functions ω(f)=1/flog(N) and $\omega^{\prime }\left(f\right)=1$, the former corresponding to the Watterson estimator. Proceeding as in the above example, the result is:

(18) $$T_{E}=\frac{S/a_{n}-\sum_{i=1}^{n-1}\frac{i}{\left(n-1\right)}\xi_{i}}{\sqrt{\mathrm{Var}\left(S/a_{n}-\sum_{j=1}^{n-1}\frac{j}{\left(n-1\right)}\xi_{j}\right)}}$$

Note that, exceptionally, the scaling of this test can also be defined by (9), without modifying the result. This is a consequence of the two equivalent forms for the weight function, Ω(f)=1/flog(N)−1 or Ω(f)=1/f−log(N).

#### 2.2.3. Weights of Singletons

The above scaling (8) is valid in principle for all weights. However, in practice there is an important exception, that is, the weight $\Omega_{1}$ of singletons. This is due to the fact that for n≪N, the number of derived singletons $\xi_{1}$ is the only estimator that is affected by very rare derived alleles (and often by sequencing errors, see [19]). More precisely, $\xi_{1}$ is actually the only estimator sensitive to the deviations from neutrality in alleles of frequency 1/N<f<1/n, which represent a vast majority of the SNPs in the population and can contain interesting biological information. Therefore, if the contribution of these alleles is relevant for the test, we can enhance (or reduce) the weight $\Omega_{1}$ by adding a factor $\Omega_{ds}$.

In the approach detailed in the previous sections, this additional contribution to $\Omega_{1}$ is needed to take into account a contribution ΔΩ(f) to Ω(f) of the form $\Delta \Omega \left(f\right)=\Omega_{ds}I\left(f<\phi \right)/\phi$ with ϕ≪1. As far as the maximum sample size never exceeds in practice $n_{max}\ll 2/\phi$, this function weights positively only alleles that appear as singletons.

Similarly, $\omega_{1}$ and $\omega_{1}^{\prime }$ can be enhanced by $\omega_{ds}$, $\omega_{ds}^{\prime }$ that correspond to contributions $\Delta \omega \left(f\right)=\omega_{ds}I\left(f<\phi \right)/\phi$, $\Delta \omega^{\prime }\left(f\right)=\omega_{ds}^{\prime }I\left(f<\phi \right)/\phi$. The test of Fu and Li [6] fall into this case.

A similar argument applies also to the weights of the number of ancestral singletons, that is, $\Omega_{n-1}$, $\omega_{n-1}$, $\omega_{n-1}^{\prime }$ that can be enhanced by factors $\Omega_{as}$, $\omega_{as}$, and $\omega_{as}^{\prime }$ respectively. However, this case is more rare, the only interesting example being the tests of Achaz [19] that avoid sequencing errors by neglecting both derived and ancestral singletons.

Summarising the results up to this section, a test $T_{\Omega }$ is completely defined by a function Ω(f) and two parameters $\Omega_{ds}$, $\Omega_{as}$ (that could depend on *n*) satisfying the conditions:

(19) $$\Omega_{ds}+\Omega_{as}+\int_{0}^{1}df\;\Omega \left(f\right)=0$$

and determining the weights through the formula:

(20) $$\Omega_{i}^{\left(n\right)}=\Omega \left(\frac{i}{n}\right)+\Omega_{ds}\delta_{i,1}+\Omega_{as}\delta_{i,n-1}-\frac{1}{n-1}\left(\Omega_{ds}+\Omega_{as}+\sum_{j=1}^{n-1}\Omega \left(\frac{j}{n}\right)\right)$$

or by a pair of functions $\omega \left(f\right),\omega^{\prime }\left(f\right)$ and parameters $\omega_{ds}$, $\omega_{ds}^{\prime }$, $\omega_{as}$, $\omega_{as}^{\prime }$ satisfying:

(21) $$\omega_{ds}+\omega_{as}+\int_{0}^{1}df\;\omega \left(f\right)=\omega_{ds}^{\prime }+\omega_{as}^{\prime }+\int_{0}^{1}df\;\omega^{\prime }\left(f\right)=1$$

and resulting in this formula for the scaling of the weights:

(22) $$\Omega_{i}^{\left(n\right)}=\frac{\omega_{ds}\delta_{i,1}+\omega_{as}\delta_{i,n-1}+\omega \left(\frac{i}{n}\right)}{\omega_{ds}+\omega_{as}+\sum_{j=1}^{n-1}\omega \left(\frac{j}{n}\right)}-\frac{\omega_{ds}^{\prime }\delta_{i,1}+\omega_{as}^{\prime }\delta_{i,n-1}+\omega^{\prime }\left(\frac{i}{n}\right)}{\omega_{ds}^{\prime }+\omega_{as}^{\prime }+\sum_{j=1}^{n-1}\omega^{\prime }\left(\frac{j}{n}\right)}$$

As showed in the examples above and below, most of the tests in the literature have this general scaling, with the only exceptions the ones contained in [8] that are not weight-consistent in the above sense.

##### Example: Fu and Li’s *F* test

This test looks for an excess of very rare derived alleles as a possible signature of negative selection [6]. The only nonzero weights are $\omega_{ds}=1$ and $\omega^{\prime }\left(f\right)=1/f\log\left(N\right)$, while $\omega \left(f\right)=\omega_{ds}^{\prime }=\omega_{as}=\omega_{as}^{\prime }=0$. The resulting test is:

(23) $$T_{F}=\frac{\xi_{1}-S/a_{n}}{\sqrt{\mathrm{Var}\left(\xi_{1}-S/a_{n}\right)}}$$

Note that this test has both singleton weights and a divergent weight function.

##### Example: Error-corrected tests of Achaz [19]

This class of tests is an attempt to correct for sequencing errors and biases in the data by removing the alleles where most of the problems manifest themselves, i.e., singletons (both ancestral and derived). With a slight generalisation of the proposal in [19], the weights of the singletons are chosen in such a way to cancel precisely the contributions of the weight functions:

(24) $$\Omega_{ds}=-\Omega \left(\frac{1}{n}\right),\Omega_{as}=-\Omega \left(1-\frac{1}{n}\right)$$

or:

(25) $$\omega_{ds}=-\omega \left(\frac{1}{n}\right),\omega_{as}=-\omega \left(1-\frac{1}{n}\right),\omega_{ds}^{\prime }=-\omega^{\prime }\left(\frac{1}{n}\right),\omega_{as}^{\prime }=-\omega^{\prime }\left(1-\frac{1}{n}\right)$$

therefore, the final weights of derived or ancestral singletons are zero. These corrections can be applied in principle to any weight function.

#### 2.2.4. Alternative Choices of Scaling

The choice of scaling discussed in the previous sections represents a quite simple and effective way to fix the dependence on *n* of a newly devised test. However, other choices are possible whose weights differ from the above ones for small *n*. The reason is that for *n* not too large, both the variance of order $f\left(1-f\right)/n≃i\left(n-i\right)/n^{3}$ in the estimation of the frequency f=i/n and the related uncertainty about how the frequencies are actually weighted in the test become important. This uncertainty originates from the (binomial) sampling of individuals from the population and there is some degree of arbitrariness in deciding how to account for it. Moreover, tests that take it into account could be inconsistent in the above sense.

A possible choice of scaling that uses the binomial sampling is the following: considering ω(f), $\omega^{\prime }\left(f\right)$ as frequency distributions, the weights $\omega_{i}$, $\omega_{i}^{\prime }$ are assigned from ω(f), $\omega^{\prime }\left(f\right)$ through the same binomial sampling that is done for allele spectra, that is:

(26) ωi=∫01dfnifi(1−f)n−iω(f)∫01df(1−fn−(1−f)n)ω(f)

(27) ωi′=∫01dfnifi(1−f)n−iω′(f)∫01df(1−fn−(1−f)n)ω′(f)

A simple example of this scaling (but with an highly divergent weight function) is given by the test for admixture [8] discussed previously. Optimal tests also follow this scaling.

##### Example: Test for admixture of Achaz [8]

This test is apparently not consistent and it does not follow the scaling (8). However, it follows another scaling related to the allele sampling. To understand this, consider the weight functions $\omega \left(f\right)=\delta \left(f-1/2\right),\omega^{\prime }\left(f\right)=1$ where δ(f−1/2) is a Dirac delta function centred in 1/2. (The Dirac delta δ(f−a) is a function whose value is 0 if f≠a and +∞ if f=a. The integral ∫δ(f−a)g(f)df is g(a) if *a* is inside the range of integration and 0 otherwise. Technically speaking it is not a mathematical function, but a distribution, i.e., an element of a dual space of regular functions.) If we scale the weights according to (26) and (27), that is:

(28) ωi=∫01dfnifi(1−f)n−iω(f)∫01df(1−fn−(1−f)n)ω(f)=ni2−n1−2−n+1

(29) ωi′=∫01dfnifi(1−f)n−iω′(f)∫01df(1−fn−(1−f)n)ω′(f)=1n−1

then the corresponding test is precisely the one proposed by Achaz. Note that the strong dependence of the test from sample size does not come only from the choice of scaling, but also from the weight function chosen, that is highly divergent.

### 2.3. Optimal Tests

#### 2.3.1. On the Existence of Generic Tests

An interesting question on the way to build good linear tests is the following: do there exist generic tests? A completely generic test for neutrality should be able to detect any deviation from the spectrum of the null model that is sufficiently large. Unfortunately, these tests do not exist. In fact, for every test defined by a set of weights $\Omega_{i}$ it is possible to find a spectrum $\xi_{i}=\alpha /ia_{n}+\left(1-\alpha \right)\Delta_{i}$ that is maximally different from the standard spectrum at least in a range of frequencies and is nevertheless undetectable by the test because its average value on this spectrum is zero. This is expressed in a more formal way in the following theorem, which shows that even the complete lack of alleles in some range of frequencies could not be always detected.

**Theorem 1.**  *For every set of n real weights $\Omega_{i}$ with $\sum_{i}\Omega_{i}=0$, there is a set of n real numbers $\Delta_{i}\neq const/i$ and a parameter α∈[0,1] that satisfy the conditions:*

(30) $$\sum_{i}i\Omega_{i}\Delta_{i}=0\;,\;\min_{i\in [1,n-1]}\left(\alpha \frac{1}{ia_{n}}+\left(1-\alpha \right)\Delta_{i}\right)=0$$

The above limitation is not a consequence of the small sample size. This can be seen for example in the framework of the scaling theory discussed in this paper. In fact, for large sample sizes, the weights can be approximated by a weight function Ω(f). In this context it is possible to prove the next theorem, that is a continuous equivalent of the previous one.

**Theorem 2.**  *For every piece-wise continuous weight function $\Omega \left(f\right)\in L_{\left[1/N,1\right]}^{1}$ such that $\int_{1/N}^{1}\Omega \left(f\right)df=0$, there is a smooth function Δ(f)≠const/f and a parameter α∈[0,1] that satisfy the conditions:*

(31) $$\int_{1/N}^{1}df\;f\Omega \left(f\right)\Delta \left(f\right)=0\;,\;\underset{f\in [0,1]}{\inf}\left(\alpha \frac{1}{f\log(N)}+\left(1-\alpha \right)\Delta \left(f\right)\right)=0$$

Note that in principle this problem can be solved using multiple tests. In fact, multiple tests should be able to detect any strong deviation from the null spectrum, provided that the number of these tests is large enough, as can be seen from the following theorem.

**Theorem 3.**  *Given at least n−2 linearly independent sets of n−1 real weights $\Omega_{i}$ with $\sum_{i}\Omega_{i}=0$, it is not possible to find a set of real numbers $\Delta_{i}\neq const/i$ such that $\sum_{i}i\Omega_{i}\Delta_{i}=0$.*

This last theorem is only a formal result and the requirement of n−2 independent tests is too strong. In practice, a small (but good) set of tests can detect most of the reasonable and interesting deviations for realistic spectra.

The above theorems can be extended to the folded spectrum. In this section and the next ones, we will consider only tests based on the unfolded spectrum. The generalisation of the discussion to the folded spectrum is usually straightforward after substituting $\xi_{i}$ (i=1…n−1) with $\eta_{i}$ (i=1…⌊n/2⌋).

#### 2.3.2. Optimal Tests and Their Geometric Structure

From the theorems of the previous section, it is clear that a single test cannot detect all the possible deviations occurring in complicated evolutionary scenarios. However, it is still possible to optimise neutrality tests for a specific alternative evolutionary scenario. A simple optimality condition has been proposed by some of the authors in [10] in order to maximise the power of the test to detect a fixed alternative scenario. We use a different notation E() and E() for the expected value with respect to the null scenario (neutral model) E() and the alternative scenario E(). If the null spectrum is $E\left(\xi_{i}\right)=\theta L\xi_{i}^{0}$ and the expected spectrum of the alternative scenario is $E\left(\xi_{i}\right)=\theta L{\bar{\xi }}_{i}$ (note that it may include an average of frequencies in a number of different related scenarios, e.g., averaging over some of the parameters of the scenario), the condition for optimal tests is the maximisation of the average result of the test under the alternative scenario:

(32) $$E\left(T_{\Omega }\right)=\frac{\sum_{i=1}^{n-1}\Omega_{i}\theta L{\bar{\xi }}_{i}/\xi_{i}^{0}}{\sqrt{\mathrm{Var}\left(\sum_{j=1}^{n-1}\Omega_{j}\xi_{j}/\xi_{j}^{0}\right)}}$$

This condition is based on the observation that the tests have mean zero and variance 1; therefore, if the distributions of the results of the tests are similar, the maximisation of the average value of the test should correspond to the maximisation of the average power of the test. It is also possible to maximise directly the power of the test, taking into account the different distribution of the results under the null and the alternative model; this possibility will be pursued in Section 3.1.

Interestingly, optimal tests show a geometric structure which becomes apparent after defining the scalar product between spectra:

(33) $$\langle \langle \xi^{\prime },\xi^{\prime \prime }\rangle \rangle \equiv \sum_{i,j}\xi_{i}^{\prime }c_{ij}^{-1}\xi_{j}^{\prime \prime }$$

where $c_{ij}^{-1}$ is the inverse of the covariance matrix $\mathrm{Cov}(\xi_{i},\xi_{j})$. Since $\mathrm{Cov}(\xi_{i},\xi_{j})$ is symmetric and positive, its inverse is also symmetric and positive, i.e., it is a positive bilinear form, therefore, the above expression defines a scalar product. Then, the optimal test for an alternative spectrum $\bar{\xi }$ can be written in the elegant form:

(34) $$T_{O}=\frac{\langle \langle \xi ,\bar{\xi }\rangle \rangle -\langle \langle \xi ,\xi^{0}\rangle \rangle \langle \langle \xi^{0},\bar{\xi }\rangle \rangle /\langle \langle \xi^{0},\xi^{0}\rangle \rangle }{\sqrt{\langle \langle \bar{\xi },\bar{\xi }\rangle \rangle -{\langle \langle \xi^{0},\bar{\xi }\rangle \rangle }^{2}/\langle \langle \xi^{0},\xi^{0}\rangle \rangle }}$$

This expression can be easily obtained as a special case of the general formula (50) that we will discuss later in the context of nonlinear tests. A direct proof of this result can be found in [10] after substituting the scalar products with the definition (33).

**Figure 2:**
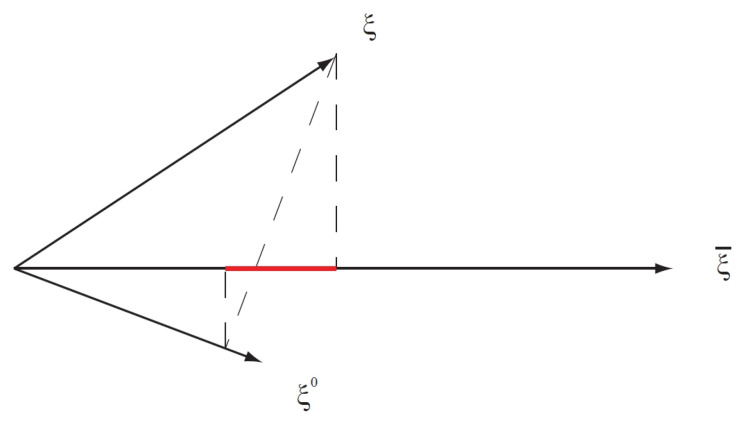
Geometrical representation of the numerator of the optimal test $T_{O}$ in (34). The length of the red line segment corresponds to the value of the numerator.

The numerator of the test is actually the matrix element between $\bar{\xi }$ and ξ of the linear operator $1-P_{\xi^{0}}$, where $P_{\xi^{0}}$ is the projection operator along $\xi^{0}$. In other words, it is proportional to the difference between the length of the projection of ξ on $\bar{\xi }$ and the length of the projection on $\bar{\xi }$ of the spectrum obtained by the projection of ξ on $\xi^{0}$, as illustrated in Figure 2.

From this geometrical interpretation it is clear that if the spectrum ξ corresponds to the null spectrum $\theta L\xi^{0}$, then the two projections are equal and the result of the test is zero. On the other side, if the spectrum is the alternative spectrum $\theta L\bar{\xi }$, then the value of the test is:

(35) $$T_{O}^{\left(max\right)}=\theta L\sqrt{\langle \langle \bar{\xi },\bar{\xi }\rangle \rangle -\frac{\langle \langle \xi^{0},\bar{\xi }\rangle \rangle^{2}}{\langle \langle \xi^{0},\xi^{0}\rangle \rangle }}$$

which is the maximum value over all possible tests in the alternative scenario. The same expression, but with a minus sign, corresponds to the minimum value.

The denominator of the test is the square root of the matrix element of the linear operator $1-P_{\xi^{0}}$ between $\bar{\xi }$ and itself. Note that both the numerator and the denominator of the test do not change by adding any (possibly negative) multiple of $\xi^{0}$ to $\bar{\xi }$, because $\xi^{0}$ lies in the kernel of $1-P_{\xi^{0}}$. This means that optimal tests depend only on the expected deviations from the null spectrum in the alternative scenario. The result of the test is maximum when the deviations of the data from the null spectrum correspond exactly to the expected ones, and it is minimum when they are opposite to the expected ones.

### 2.4. Beyond Linear Neutrality Tests

#### 2.4.1. Quadratic and Nonlinear Tests

Almost all the neutrality tests proposed in the literature are linear in the spectrum $\xi_{i}$. As far as we know, there is only one exception, namely the $G_{\xi }$ test of Fu [11]. This test is a quadratic polynomial reminiscent of Hotelling’s $t^{2}$ statistics for the different components of the spectra:

(36) $$G=\sum_{i,j=1}^{n-1}c_{ij}^{-1}\left(\xi_{i}-\theta L\xi_{i}^{0}\right)\left(\xi_{j}-\theta L\xi_{j}^{0}\right)$$

where $c_{ij}^{-1}$ is the inverse of the covariance matrix $\mathrm{Cov}(\xi_{i},\xi_{j})$. Actually, the test proposed by Fu is an approximation to this test with a different normalisation, namely:

(37) $$G_{\xi }=\frac{1}{n-1}\sum_{i=1}^{n-1}\frac{{\left(\xi_{i}-\theta L\xi_{i}^{0}\right)}^{2}}{\mathrm{Var}\left(\xi_{i}\right)}$$

In this approximation, the off diagonal terms in the covariance can be neglected [9,11]. For large samples, the distribution of the results of the test *G* tends to a $\chi^{2}$ distribution with n−1 degrees of freedom.

Fu’s approach cannot be extended to general quadratic or higher order tests, because the distribution of the results of the test would be generally unknown and not positive definite. For this reason we propose to rescale the tests to have zero mean and variance 1. With this normalisation, we expect that the distribution would asymptotically converge to a Gaussian N(0,1) for all tests. As an example, the (re)normalised version of Fu’s test would be:

(38) $$T_{G}=\frac{\sum_{i,j=1}^{n-1}c_{ij}^{-1}\left(\xi_{i}-\theta L\xi_{i}^{0}\right)\left(\xi_{j}-\theta L\xi_{j}^{0}\right)-\left(n-1\right)}{\sqrt{\mathrm{Var}\left(\sum_{i,j=1}^{n-1}c_{ij}^{-1}\left(\xi_{i}-\theta L\xi_{i}^{0}\right)\left(\xi_{j}-\theta L\xi_{j}^{0}\right)-\left(n-1\right)\right)}}$$

Since the only difference between this test and the original one is the normalisation and a shift by a constant factor n−1, the power of the test is the same.

Now we present a systematic discussion of nonlinear tests that are generic polynomials (or eventually power series) in the spectrum $\xi_{i}$. All the tests are rescaled to be centred (i.e., to have zero mean) and have variance 1. We denote by $\mu_{ijk\ldots }$ the moments of the spectrum under the null model, that is, $\mu_{ijk\ldots }=E\left(\xi_{i}\xi_{j}\xi_{k}\ldots \right)$. With this definition, $\mu_{i}=\theta L\xi_{i}^{0}$. Note that all these moments depend on θ. In the approximation of unlinked (independent) sites and small θ, the second moments are equal to $\mu_{ij}=\theta L\xi_{i}^{0}\delta_{ij}+\theta^{2}L^{2}\xi_{i}^{0}\xi_{j}^{0}$.

The weights of general nonlinear tests can depend explicitly on θ, as seen in the previous example. To compute the values of the tests, the (unknown) parameter θ is substituted with an estimator $\hat{\theta }$. Unlike the linear case, in this case there are two different classes of tests, related to the dependence on $\hat{\theta }$ of the centredness: *strongly centred* and *weakly centred* tests.

Strongly centred tests are tests that are always centred for any value of $\hat{\theta }$, even if it is different from the actual value of θ. The general form for strongly centred tests is:

(39) $$T_{\Omega }=\frac{\sum_{i=1}^{n-1}\Omega_{i}^{\left(1\right)}\xi_{i}+\sum_{i,j=1}^{n-1}\Omega_{ij}^{\left(2\right)}\xi_{i}\xi_{j}+\sum_{i,j,k=1}^{n-1}\Omega_{ijk}^{\left(3\right)}\xi_{i}\xi_{j}\xi_{k}+\cdots }{\sqrt{\mathrm{Var}\left(\sum_{i=1}^{n-1}\Omega_{i}^{\left(1\right)}\xi_{i}+\sum_{i,j=1}^{n-1}\Omega_{ij}^{\left(2\right)}\xi_{i}\xi_{j}+\sum_{i,j,k=1}^{n-1}\Omega_{ijk}^{\left(3\right)}\xi_{i}\xi_{j}\xi_{k}+\cdots \right)}}$$

with the real symmetric weights $\Omega_{ijk\ldots }^{\left(n\right)}$ satisfying the set of conditions:

(40) $$0=\sum_{i=1}^{n-1}\Omega_{i}^{\left(1\right)}\mu_{i}^{\left(m\right)}+\sum_{i,j=1}^{n-1}\Omega_{ij}^{\left(2\right)}\mu_{ij}^{\left(m\right)}+\ldots \;,\;m=1,2,3\ldots$$

where we denote by $\mu_{ijk\ldots }^{\left(p\right)}$ the *p*-th term of the Taylor expansion with respect to θL of $\mu_{ijk\ldots }$ (in other words, $\mu_{ijk\ldots }=\sum_{p}\theta^{p}L^{p}\mu_{ijk\ldots }^{\left(p\right)}$, where the coefficients $\mu_{ijk\ldots }^{\left(p\right)}$ are independent on θ). The sum can be limited to polynomials of some finite order in $\xi_{i}$ or it can be a (convergent) power series. If we introduce the notation I=ijk… to denote a group of $n_{I}$ indices, we can rewrite the test in the simpler form:

(41) $$T_{\Omega }=\frac{\sum_{I}\Omega_{I}^{\left(n_{I}\right)}{\left(\xi \ldots \xi \right)}_{I}}{\sqrt{\mathrm{Var}\left(\sum_{I}\Omega_{I}^{\left(n_{I}\right)}{\left(\xi \ldots \xi \right)}_{I}\right)}}$$

with the conditions:

(42) $$0=\sum_{I}\Omega_{I}^{\left(n_{I}\right)}\mu_{I}^{\left(m\right)}\;,\;m=1,2,3\ldots$$

If we constrain these tests to be first order polynomials in $\xi_{i}$, we recover the linear case with $\Omega_{i}^{\left(1\right)}=\Omega_{i}/\xi_{i}^{0}$. Note that linear tests are always strongly centred. In fact, in the infinite site model the spectrum is always proportional to θ, which consequently factorises out by linearity and therefore has no effect on the centredness.

Weakly centred tests are tests that are centred but not strongly centred, i.e., they are centred if and only if $\hat{\theta }=\theta$. The general form for weakly centred tests is:

(43) $$T_{\Gamma }=\frac{\gamma +\sum_{i=1}^{n-1}\Gamma_{i}^{\left(1\right)}\xi_{i}+\sum_{i,j=1}^{n-1}\Gamma_{ij}^{\left(2\right)}\xi_{i}\xi_{j}+\sum_{i,j,k=1}^{n-1}\Gamma_{ijk}^{\left(3\right)}\xi_{i}\xi_{j}\xi_{k}+\cdots }{\sqrt{\mathrm{Var}\left(\gamma +\sum_{i=1}^{n-1}\Gamma_{i}^{\left(1\right)}\xi_{i}+\sum_{i,j=1}^{n-1}\Gamma_{ij}^{\left(2\right)}\xi_{i}\xi_{j}+\sum_{i,j,k=1}^{n-1}\Gamma_{ijk}^{\left(3\right)}\xi_{i}\xi_{j}\xi_{k}+\cdots \right)}}$$

with the condition:

(44) $$0=\gamma +\sum_{i=1}^{n-1}\Gamma_{i}^{\left(1\right)}\mu_{i}+\sum_{i,j=1}^{n-1}\Gamma_{ij}^{\left(2\right)}\mu_{ij}+\sum_{i,j,k=1}^{n-1}\Gamma_{ijk}^{\left(3\right)}\mu_{ijk}+\ldots$$

where the $\Gamma_{ijk\ldots }$ are real symmetric weights, possibly dependent on θ. We can simplify these expressions using the same notation as above, obtaining the simpler form:

(45) $$T_{\Gamma }=\frac{\gamma +\sum_{I}\Gamma_{I}^{\left(n_{I}\right)}{\left(\xi \ldots \xi \right)}_{I}}{\sqrt{\mathrm{Var}\left(\gamma +\sum_{I}\Gamma_{I}^{\left(n_{I}\right)}{\left(\xi \ldots \xi \right)}_{I}\right)}}$$

with the condition:

(46) $$0=\gamma +\sum_{I}\Gamma_{I}^{\left(n_{I}\right)}\mu_{I}$$

Additionally, for this class of tests the sum can be limited to polynomials of fixed order or extended to power series. Note that the rescaled version of the *G* test by Fu presented above belongs to this class.

The important difference between strongly and weakly centred tests is related to the robustness with respect to a biased estimation of θ. Since the class of weakly centred tests is much larger than the class of strongly centred ones, it should be easier to find powerful tests in the former class than in the latter. However, even if weakly centred tests could be more powerful, they would not be centred in scenarios where the value of θ could not be estimated precisely. On the other side, strongly centred tests are robust with respect to a bad estimation of θ and therefore they would be preferable in scenarios where an unbiased estimation of θ is troublesome.

The scaling rule (8) can be generalised to nonlinear tests in terms of functions $\Omega^{\left(n_{I}\right)}\left(f_{1},f_{2}\ldots f_{n_{I}}\right)$ for strongly centred and $\Gamma^{\left(n_{I}\right)}\left(f_{1},f_{2}\ldots f_{n_{I}}\right)$ for weakly centred tests:

(47) $$\Omega_{I}^{\left(n_{I}\right)}≃\frac{1}{n^{n_{I}}}\Omega^{\left(n_{I}\right)}\left(\frac{i}{n},\frac{j}{n},\frac{k}{n}\ldots \right)$$

(48) $$\Gamma_{I}^{\left(n_{I}\right)}≃\frac{1}{n^{n_{I}}}\Gamma^{\left(n_{I}\right)}\left(\frac{i}{n},\frac{j}{n},\frac{k}{n}\ldots \right)$$

Fixing the precise scaling is more ambiguous than in the linear case because there are many different ways to preserve centredness. For this reason, the choice of scaling would be different for strongly and weakly centred tests and will not be discussed here.

All the possible nonlinear neutrality tests based on the frequency spectrum fall into one of the two classes presented in this section and have the form (41) and (42), or (45) and (46). Since both these classes contain an infinite number of possible choices of weights, the only reasonable criterion to study general nonlinear tests is to select the most powerful or interesting ones. Apart from the Hotelling choice of Fu [11], the most interesting choice is apparently the subclass of nonlinear optimal tests, which will be discussed in the next sections.

#### 2.4.2. Strongly Centred Optimal Tests

As discussed for the linear case, optimal tests depend on the expected alternative scenario. In the nonlinear case, in principle it would be possible to find generic optimal tests, but there is no clear framework to obtain them. For this reason we limit our study to the case of optimal tests for a specific alternative scenario. We denote by ${\bar{\mu }}_{ijk\ldots }=E\left(\xi_{i}\xi_{j}\xi_{k}\ldots \right)$ the moments of the alternative spectrum for this scenario.

Since we use the same normalisation for linear and nonlinear tests, the optimality condition corresponds to the maximisation of the expected value of the test under the alternative scenario:

(49) $$E\left(T_{\Omega }\right)=\frac{\sum_{I}\Omega_{I}^{\left(n_{I}\right)}{\bar{\mu }}_{I}}{\sqrt{\mathrm{Var}\left(\sum_{I}\Omega_{I}^{\left(n_{I}\right)}{\left(\xi \ldots \xi \right)}_{I}\right)}}$$

and can be justified as in the linear case.

We denote by $\tilde{I}$ the ordered sequence of the indices contained in I=ijk… and by $\sigma \left(I\right)$ the number of distinct permutations of the sequence I, i.e., the total number of permutations divided by the number of permutations that leave I invariant. The main result for the optimal weights is presented in this theorem.

**Theorem 4.**  *The maxima of $E(T_{\Omega })$ correspond to the weights:*

(50) $$\Omega_{I}^{\left(n_{I}\right)}=\frac{1}{\sigma \left(I\right)}\left(\sum_{\tilde{L}}C_{\tilde{I}\tilde{L}}^{-1}{\bar{\mu }}_{\tilde{L}}-\sum_{k}\sum_{l}\sum_{\tilde{L}}C_{\tilde{I}\tilde{L}}^{-1}\mu_{\tilde{L}}^{\left(k\right)}M_{kl}\sum_{\tilde{J},\tilde{K}}\mu_{\tilde{J}}^{\left(l\right)}C_{\tilde{J}\tilde{K}}^{-1}{\bar{\mu }}_{\tilde{K}}\right)$$

*where the matrices $C_{\tilde{I}\tilde{J}}^{-1}$ and $M_{kl}$ satisfy the identities:*

(51) $$\sum_{\tilde{K}}C_{\tilde{I}\tilde{K}}^{-1}\left(\mu_{\tilde{K}\tilde{J}}-\mu_{\tilde{K}}\mu_{\tilde{J}}\right)=\delta_{\tilde{I}\tilde{J}}$$

(52) $$\sum_{r}M_{kr}\sum_{\tilde{I},\tilde{L}}\mu_{\tilde{I}}^{\left(r\right)}C_{\tilde{I}\tilde{L}}^{-1}\mu_{\tilde{L}}^{\left(l\right)}=\delta_{kl}$$

*Moreover, the variance of the corresponding unnormalised test under the null model is equal to its expected value under the alternative model:*

(53) $$\mathrm{Var}\left(\sum_{I}\Omega_{I}^{\left(n_{I}\right)}{\left(\xi \ldots \xi \right)}_{I}\right)=\sum_{I}\Omega_{I}^{\left(n_{I}\right)}{\bar{\mu }}_{I}$$

Note that in general all the weights of the above optimal solution (50) are nonzero, therefore the maximum average value of the test for optimal tests built on polynomials of degree *d* increases with the degree *d*. This suggests that optimal tests of higher degree should be more powerful than linear optimal tests.

We provide explicit formulae for the above weights for the optimal quadratic test in the independent sites approximation. Given $E\left(\xi_{i}\right)=\mu_{i}$ and $E\left(\xi_{i}\right)={\bar{\mu }}_{i}$, the relevant weights $\Omega_{I}^{\left(n_{I}\right)}$ are:

(54) $$\begin{array}{cc} \Omega_{i}^{\left(1\right)} & =\left(\Sigma_{\mu }+2-\Sigma_{\bar{\mu }}\right)\left(\frac{{\bar{\mu }}_{i}}{\mu_{i}}-\frac{\Sigma_{\bar{\mu }}}{\Sigma_{\mu }}\right)-\frac{1}{2}\left(\frac{{\bar{\mu }}_{i}^{2}}{\mu_{i}^{2}}-\frac{\Sigma_{\bar{\mu }}^{2}}{\Sigma_{\mu }^{2}}\right) \end{array}$$

(55) $$\begin{array}{cc} \Omega_{ii}^{\left(2\right)} & =-\left(\frac{{\bar{\mu }}_{i}}{\mu_{i}}-\frac{\Sigma_{\bar{\mu }}}{\Sigma_{\mu }}\right)+\frac{1}{2}\left(\frac{{\bar{\mu }}_{i}^{2}}{\mu_{i}^{2}}-\frac{\Sigma_{\bar{\mu }}^{2}}{\Sigma_{\mu }^{2}}\right) \end{array}$$

(56) $$\begin{array}{cc} \Omega_{ij}^{\left(2\right)} & =\frac{1}{2}\left[\left(\frac{{\bar{\mu }}_{i}{\bar{\mu }}_{j}}{\mu_{i}\mu_{j}}-\frac{\Sigma_{\bar{\mu }}^{2}}{\Sigma_{\mu }^{2}}\right)-\left(\frac{{\bar{\mu }}_{i}}{\mu_{i}}-\frac{\Sigma_{\bar{\mu }}}{\Sigma_{\mu }}\right)-\left(\frac{{\bar{\mu }}_{j}}{\mu_{j}}-\frac{\Sigma_{\bar{\mu }}}{\Sigma_{\mu }}\right)\right] \end{array}$$

where $\Sigma_{\mu }=\sum_{i=1}^{n-1}\mu_{i}$ and $\Sigma_{\bar{\mu }}=\sum_{i=1}^{n-1}{\bar{\mu }}_{i}$. All these formulae are also valid for the folded spectrum if the appropriate $\mu_{i}$ and ${\bar{\mu }}_{i}$ are used. These results are discussed in Appendix A.7.

For optimal tests of higher degree, explicit expressions become cumbersome and the numerical implementation of the test (50) and the matrices (51) and (52) is more convenient.

#### 2.4.3. Weakly Centred Optimal Tests

In this case the optimality condition corresponds to the maximisation of the expression:

(57) $$E\left(T_{\Gamma }\right)=\frac{\gamma +\sum_{I}\Gamma_{I}^{\left(n_{I}\right)}{\bar{\mu }}_{I}}{\sqrt{\mathrm{Var}\left(\gamma +\sum_{I}\Gamma_{I}^{\left(n_{I}\right)}{\left(\xi \ldots \xi \right)}_{I}\right)}}$$

with the same condition:

(58) $$0=\gamma +\sum_{I}\Gamma_{I}^{\left(n_{I}\right)}\mu_{I}$$

The simplest case corresponds to a first order polynomial:

(59) $$T_{\Gamma }=\frac{\gamma +\sum_{i=1}^{n-1}\Gamma_{i}^{\left(1\right)}\xi_{i}}{\sqrt{\gamma^{2}+2\gamma \sum_{j=1}^{n-1}\Gamma_{j}^{\left(1\right)}\mu_{j}+\sum_{j=1}^{n-1}\sum_{k=1}^{n-1}\Gamma_{j}^{\left(1\right)}\Gamma_{k}^{\left(1\right)}\mu_{jk}}}$$

whose maximum corresponds to the optimal weights:

(60) $$\Gamma_{i}^{\left(1\right)}=\sum_{j=1}^{n-1}c_{ij}^{-1}\left({\bar{\mu }}_{j}-\mu_{j}\right)\;,\;\gamma =-\sum_{j=1}^{n-1}\sum_{k=1}^{n-1}\mu_{j}c_{jk}^{-1}\left({\bar{\mu }}_{k}-\mu_{k}\right)$$

where $c_{ij}^{-1}$ is the inverse matrix of the covariance matrix $c_{ij}=\mu_{ij}-\mu_{i}\mu_{j}$. Since γ≠0 for this optimal test, the value of this test for the specific scenario for which it is built is larger than the value of the corresponding linear optimal test. In fact the maximum of the test is:

(61) $$E\left(T_{\Gamma }\right)=\sqrt{\sum_{j=1}^{n-1}\sum_{k=1}^{n-1}\left({\bar{\mu }}_{j}-\mu_{j}\right)c_{jk}^{-1}\left({\bar{\mu }}_{k}-\mu_{k}\right)}$$

that should be compared to the maximum of the optimal test for the linear case, which can be rewritten as:

(62) $$E{\left(T_{\Omega }\right)}_{linear}=\sqrt{\sum_{j=1}^{n-1}\sum_{k=1}^{n-1}\left({\bar{\mu }}_{j}-\mu_{j}\right)c_{jk}^{-1}\left({\bar{\mu }}_{k}-\mu_{k}\right)-\frac{{\left(\sum_{j=1}^{n-1}\sum_{k=1}^{n-1}\mu_{j}c_{jk}^{-1}\left({\bar{\mu }}_{k}-\mu_{k}\right)\right)}^{2}}{\sum_{j=1}^{n-1}\sum_{k=1}^{n-1}\mu_{j}c_{jk}^{-1}\mu_{k}}}$$

The comparison shows clearly that nonlinear optimal tests are always more powerful than linear optimal tests for the same scenario.

The form of the results for the general case is similar to this simple case.

**Theorem 5.**  *The maxima of $E(T_{\Gamma })$ correspond to the weights:*

(63) $$\Gamma_{I}^{\left(n_{I}\right)}=\frac{1}{\sigma \left(I\right)}\sum_{\tilde{J}}C_{\tilde{I}\tilde{J}}^{-1}\left({\bar{\mu }}_{\tilde{J}}-\mu_{\tilde{J}}\right)\;,\;\gamma =-\sum_{\tilde{J}}\mu_{\tilde{J}}\sum_{\tilde{K}}C_{\tilde{J}\tilde{K}}^{-1}\left({\bar{\mu }}_{\tilde{K}}-\mu_{\tilde{K}}\right)$$

*where $C_{\tilde{I}\tilde{J}}^{-1}$ satisfied the identity:*

(64) $$\sum_{\tilde{K}}C_{\tilde{I}\tilde{K}}^{-1}\left(\mu_{\tilde{K}\tilde{J}}-\mu_{\tilde{K}}\mu_{\tilde{J}}\right)=\delta_{\tilde{I}\tilde{J}}$$

*Moreover, the variance of the corresponding unnormalised test under the null model is equal to its expected value under the alternative model:*

(65) $$\mathrm{Var}\left(\gamma +\sum_{I}\Gamma_{I}^{\left(n_{I}\right)}{\left(\xi \ldots \xi \right)}_{I}\right)=\sum_{I}\Gamma_{I}^{\left(n_{I}\right)}{\bar{\mu }}_{I}+\gamma$$

Also in this case, the power of optimal tests based on polynomials of higher degree increases with the degree of the polynomial.

It is possible to give explicit expressions of the above matrix and moments for the optimal quadratic test. The formulae for the weights $\Gamma_{I}^{\left(n_{I}\right)}$ for the unfolded spectrum are:

(66) $$\begin{array}{cc} \Gamma_{i}^{\left(1\right)} & =\left(\Sigma_{\mu }+2-\Sigma_{\bar{\mu }}\right)\left(\frac{{\bar{\mu }}_{i}}{\mu_{i}}-1\right)-\frac{1}{2}\left(\frac{{\bar{\mu }}_{i}^{2}}{\mu_{i}^{2}}-1\right) \end{array}$$

(67) $$\begin{array}{cc} \Gamma_{ii}^{\left(2\right)} & =\frac{1}{2}{\left(\frac{{\bar{\mu }}_{i}}{\mu_{i}}-1\right)}^{2} \end{array}$$

(68) $$\begin{array}{cc} \Gamma_{ij}^{\left(2\right)} & =\frac{1}{2}\left(\frac{{\bar{\mu }}_{i}}{\mu_{i}}-1\right)\left(\frac{{\bar{\mu }}_{j}}{\mu_{j}}-1\right) \end{array}$$

(69) $$\begin{array}{cc} \gamma & =\frac{1}{2}\left(\Sigma_{\mu }-\Sigma_{\bar{\mu }}\right)\left(\Sigma_{\mu }+2-\Sigma_{\bar{\mu }}\right) \end{array}$$

These results are valid in the independent sites approximation. They are also valid for the folded spectrum if the appropriate $\mu_{i}$ and ${\bar{\mu }}_{i}$ are used. An expression for the denominator of the test in the independent sites approximation can be found in Appendix A.7.

### 2.5. Empirical Simulations and Code Used for Studying the Statistical Power of Neutrality Tests

We obtained the frequency spectrum of samples for different subdivision and expansion models using one million iterations. Simulation parameters, as well as all the data frequencies from the simulations are included in Zenodo (DOI: 10.5281/zenodo.8279694). Linear and nonlinear tests were calculated using our own code, also available in the same link. Additionally, linear and nonlinear optimal tests are also included in the program *mstatspop* (https://github.com/cragenomica/mstatspop, accessed on 15 August 2023), to facilitate the calculation of these tests by the users.

## 3. Results

### 3.1. General Optimisation of Linear Tests

The condition for optimal tests is the maximisation of $E(T_{\Omega })$ under the alternative scenario. However, a better approach would by the maximisation of the power of the test to reject the neutral model in the alternative scenario, given a choice of significance level α. This approach requires the knowledge of the form of the probability distributions $p(T_{\Omega }=t|H_{0})$, $p(T_{\Omega }=t|H_{1})$ where $H_{0}$ and $H_{1}$ are the null and alternative model, or equivalently of all the moments of the spectrum $E(\xi_{i}\xi_{j}\xi_{k}\ldots )$ and $E(\xi_{i}\xi_{j}\xi_{k}\ldots )$.

Since this information is usually not available in analytic form and hard to obtain computationally, we limit to the case where the distribution can be well approximated by a Gaussian both for the null and for the expected model. Then, the only information needed are the spectra $\mu_{i}=E\left(\xi_{i}\right)$, ${\bar{\mu }}_{i}=E\left(\xi_{i}\right)$ and their covariance matrices $c_{ij}=E\left(\xi_{i}\xi_{j}\right)-E\left(\xi_{i}\right)E\left(\xi_{j}\right)$, ${\bar{c}}_{ij}=E\left(\xi_{i}\xi_{j}\right)-E\left(\xi_{i}\right)E\left(\xi_{j}\right)$.

We expect that both in this approximation and in the general case, the tests with maximum power will depend on the significance level chosen, therefore limiting the interest of these test and the possibilities of comparison between results of the test on samples from different experiments.

We call $\tau ={\mathrm{erf}}^{-1}\left(1-2\alpha \right)$ the *z*-value corresponding to the critical *p*-value α. In the Gaussian approximation, the power is given by the following expression:

(70) $$\mathrm{Power}=\frac{1}{2}\left[1+\mathrm{erf}\left(\frac{\sum_{j}{\bar{\mu }}_{j}\Omega_{j}-\tau \sqrt{\sum_{j,k}c_{jk}\Omega_{j}\Omega_{k}}}{\sqrt{\sum_{j,k}{\bar{c}}_{jk}\Omega_{j}\Omega_{k}}}\right)\right]$$

then its maximisation is equivalent to the maximisation of:

(71) $$\frac{\sum_{j}{\bar{\mu }}_{j}\Omega_{j}-\tau \sqrt{\sum_{j,k}c_{jk}\Omega_{j}\Omega_{k}}}{\sqrt{\sum_{j,k}{\bar{c}}_{jk}\Omega_{j}\Omega_{k}}}$$

In the general case, the weights corresponding to the maximum depend explicitly on τ and therefore on α. This dependence is expected but unwanted, since the interpretation of the test depends explicitly on the critical *p*-value chosen.

There is only one case with weights independent on τ, that is the case of ${\bar{c}}_{ij}$ (approximately) proportional to $c_{ij}$. In this case the maximisation of the power of the test is (approximately) equivalent to the maximisation of the average result of the test, which is precisely the condition for optimal tests in the sense of [10]. In fact, in this case, optimal tests correspond precisely to an approximation of the likelihood-ratio tests under the assumption of Gaussian likelihood functions, and are therefore approximately the most powerful tests because of the Neyman–Pearson lemma.

As a side note, there is also a regime of values of α such that the weights corresponding to maximum power are independent of α, that is, the regime τ(α)≫1. In this case the power is an increasing function of $\sum_{j,k}{\bar{c}}_{jk}\Omega_{j}\Omega_{k}/\sum_{l,m}c_{lm}\Omega_{l}\Omega_{m}$ and the weights are simply given by the null eigenvector (or linear combination of null eigenvectors) of the matrix ${\bar{c}}_{ij}-\chi c_{ij}$, where χ is uniquely defined by the requirement that ${\bar{c}}_{ij}-\chi c_{ij}$ be a negative semidefinite matrix with at least a null eigenvalue. However, this regime is uninteresting because such small significance levels are practically useless (if τ∼10, the corresponding critical *p*-value is $\alpha ∼10^{-20}$). However, it could be possible to build interesting tests with higher power by selecting linear combinations of the weights of the two α-independent tests discussed above, that is, optimal tests and tests that maximise the alternative/null variance ratio $\sum_{j,k}{\bar{c}}_{jk}\Omega_{j}\Omega_{k}/\sum_{l,m}c_{lm}\Omega_{l}\Omega_{m}$.

#### 3.1.1. Generalised Optimality Conditions

In a more general linear framework, the (normalised) test has the form $T=\sum_{i=1}^{n-1}\Omega_{i}\xi_{i}/\xi_{i}^{0}$. We denote its moments under the standard neutral model (SNM) and the alternative model by:

(72) $$E_{0}=E{\left(T\right)}_{SNM}=\theta L\sum_{i=1}^{n-1}\Omega_{i}\;,\;V_{0}=\mathrm{Var}{\left(T\right)}_{SNM}=\sum_{i,j=1}^{n-1}\Omega_{i}\Omega_{j}c_{ij}/\xi_{i}^{0}\xi_{j}^{0}$$

(73) $$E=E{\left(T\right)}_{alt}=\theta L\sum_{i=1}^{n-1}\Omega_{i}{\bar{\xi }}_{i}/\xi_{i}^{0}\;,\;V=\mathrm{Var}{\left(T\right)}_{alt}=\sum_{i,j=1}^{n-1}\Omega_{i}\Omega_{j}{\bar{c}}_{ij}/\xi_{i}^{0}\xi_{j}^{0}$$

where $E{\left(\xi_{i}\right)}_{SNM}=\theta L\xi_{i}^{0}$, $E{\left(\xi_{i}\right)}_{alt}=\theta L{\bar{\xi }}_{i}$, $\mathrm{Cov}{\left(\xi_{i},\xi_{j}\right)}_{SNM}=c_{ij}$ and $\mathrm{Cov}{\left(\xi_{i},\xi_{j}\right)}_{alt}={\bar{c}}_{ij}$. For the standard neutral model $\xi_{i}^{0}=1/i$.

The optimality condition can be the maximisation of a general function $M(E_{0},V_{0},E,V)$ of these quantities with respect to the weights $\Omega_{i}$. Since we are dealing with linear combination of the frequency spectrum with mean $E_{0}=0$ and variance $V_{0}=1$, the function effectively depends on E,V only. Maximisation of power (in the Gaussian approximation), given a significance level α, is equivalent to the maximisation of:

(74) $$M\left(E,V\right)=\frac{E-\Phi^{-1}\left(1-\alpha \right)}{\sqrt{V}}$$

If there is some knowledge of the variance of the alternative spectrum, or at least of the contribution for unlinked sites, maximisation of power is not the only possible optimisation. In other words, it is possible to optimise with respect to other functions M(E,V) of the alternative mean E and variance V of the test, in order to obtain, e.g., more robust or conservative tests.

For example:

- “Optimal tests”: if V is assumed to be completely unknown, the best we can do is minimise the *p*-value of the expected alternative spectrum, that is, in the Gaussian approximation $p=1-\Phi (\left(E-E_{0}\right)/\sqrt{V_{0}})$ where Φ(z) is the cumulative distribution function for the standard normal distribution. This is equivalent to the maximisation of:
  (75) $$M\left(E_{0},V_{0},E,V\right)=\frac{E-E_{0}}{\sqrt{V_{0}}}$$
- Most powerful one-tail tests: in the Gaussian approximation, the power of the right tail of test to reject the neutral Wright–Fisher model given a significance level α is $\mathrm{Power}=1-\Phi (\left(E_{0}+\tau \sqrt{V_{0}}-E\right)/\sqrt{V})$, where $\tau =\Phi^{-1}\left(1-\alpha \right)$. Maximising this power is equivalent to the maximisation of:
  (76) $$M\left(E_{0},V_{0},E,V\right)=\frac{E-E_{0}-\tau \sqrt{V_{0}}}{\sqrt{V}}$$
  If $V\propto V_{0}$, this is equivalent to the previous case and we retrieve the optimal tests of [10] that are therefore the most powerful tests in this approximation.
- Most powerful two-tails tests: in the Gaussian approximation, the power of both tails of the test to reject the neutral Wright–Fisher model is:
  (77) $$M\left(E_{0},V_{0},E,V\right)=1-\Phi \left(\frac{E_{0}+\tau^{\prime }\sqrt{V_{0}}-E}{\sqrt{V}}\right)+\Phi \left(\frac{E_{0}-\tau^{\prime }\sqrt{V_{0}}-E}{\sqrt{V}}\right)$$
  where $\tau^{\prime }=\Phi^{-1}\left(1-\alpha /2\right)$ and α is the significance level. If $E_{0}=0$, this maximisation is equivalent to the maximisation of $\left(E-E_{0}-\tau^{\prime }\sqrt{V_{0}}\right)/\sqrt{V}$ similar to the previous case.
- Penalisations for high or low variance, depending on an extra parameter ν, such as:
  (78) $$M\left(E_{0},V_{0},E,V\right)=\frac{E-E_{0}}{{\left(\sqrt{V_{0}}+\sqrt{V}\right)}^{\nu }}$$

#### 3.1.2. Tunable Optimal Tests

Interestingly, it turns out that the choice of an optimisation criterion—i.e., of a function M(E,V) to be maximised—can be performed simply by tuning a parameter λ in a simple class of tests that we call “tunable optimal tests”.

The family of optimised tests with tunable parameter λ has the simple form:

(79) $$T=\frac{\sum_{i=1}^{n-1}i\Omega_{i}\left(\lambda \right)\xi_{i}}{\sqrt{\mathrm{Var}(\sum_{i=1}^{n-1}i\Omega_{i}\left(\lambda \right)\xi_{i})}}$$

with weights:

(80) $$\Omega_{i}\left(\lambda \right)=\frac{1}{i}\left[1-\frac{a_{n}}{1+\lambda i{\bar{\xi }}_{i}}{\left(\sum_{j=1}^{n-1}\frac{1}{j(1+\lambda j{\bar{\xi }}_{j})}\right)}^{-1}\right]$$

where $a_{n}=\sum_{j=1}^{n-1}1/j$.

The interest of this family of tests lies in the following property: for every possible choice of the optimisation function M(E,V), the test corresponding to the maximum value of this function belongs to this family (see Appendix B).

Usual optimal tests [10] are obtained from the maximisation of M(E,V)=E and correspond to $\lambda =0^{+}$, that is, to the usual weights $\Omega_{i}={\bar{\xi }}_{i}-\frac{1}{i}\frac{\sum_{j=1}^{n-1}{\bar{\xi }}_{j}}{a_{n}}$.

More generally, λ can be obtained directly by evaluating E, V with the weights (80) and then looking for the maximisation of M(E(λ),V(λ)). Note that there could be several local maxima.

This class of tests is simple but far more flexible than usual optimal tests. However, note that the exact choice of weights depends explicitly on θ and α. Furthermore, the smoothness of the choice of weights with respect to the evolutionary parameters is not assured. In principle, a slight change of evolutionary scenario could change the optimal weights abruptly.

### 3.2. Simulations of the Power of Optimal Tests

Since a theoretical evaluation of the power of optimal tests of different degree is not possible, we evaluate numerically the power of some of these tests in different scenarios. We consider the best possible case, that is, we assume that the precise value of θ is known. Moreover we assume unlinked sites and θ≪1. In this approximation, as shown in Appendix A.7, the moments $E(\xi_{i}\xi_{j}\xi_{k}\ldots )$ depend only on the first moments $\mu_{i}=\theta L\xi_{i}^{0}$ and similarly $E(\xi_{i}\xi_{j}\xi_{k}\ldots )$ depend only on ${\bar{\mu }}_{i}=\theta L{\bar{\xi }}_{i}$, therefore optimal tests depend only on the alternative and null average spectra.

Note that for numerical simulations of optimal tests of higher degree, the numerical implementation can be made easier if all the occurrences of inverse covariance matrices $C_{\tilde{I}\tilde{J}}^{-1}$ in the the above formulae are replaced with the corresponding second moments $\mu_{\tilde{I}\tilde{J}}^{-1}$, both in the expressions (50), (63), and in the definition (52). The test is the same because of the centredness condition, as it can be verified explicitly.

We compare four optimal tests. The first two are the linear and quadratic strongly centred optimal tests, which are denoted by $T_{O(1)}^{sc}$ and $T_{O(2)}^{sc}$ respectively. The third test is the weakly centred optimal test $T_{O(1)}^{wc}$ based on a first order polynomial and presented in (59). The last optimal test $T_{O(2)}^{wc}$ is also weakly centred and based on on a quadratic polynomial. The explicit formulae for the computation of the weights of $T_{O(2)}^{sc}$ and $T_{O(2)}^{wc}$ were given in Equations (54)–(56) and (66)–(69).

We simulated two demographic processes: (A) subdivision, where two populations having identical size exchange individuals given a symmetric migration rate *M*, then individuals are sampled from one population only; (B) expansion, where the population size changes by a factor $N_{0}/N=10$ at a time *T* before present (in units of 4N generations). For each value of the parameters *M* and *T*, $10^{6}$ simulations were performed with *mlcoalsim* v1.98b [20] for a region of 1000 bases with variability θ=0.05 and recombination ρ=∞ and a sample size of n=20 (haploid) individuals. Confidence intervals at 95% level were estimated from $10^{6}$ simulations of the standard neutral coalescent with the same parameters.

**Figure 3:**
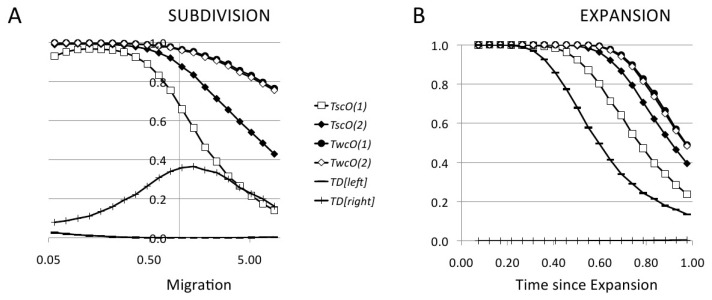
Statistical power of nonlinear optimal tests from coalescent simulations for the 5% tail, compared with Tajima’s *D* test (for the left and the right tail). The parameters for the simulations are: n=20, θ=0.05, L=1000 bp, ρ=∞; two populations considered but only one sampled (for panel **A**); expansion factor $N_{0}/N=10$ (for panel **B**).

In Figure 3 we compare the power of the tests in the best possible situation, namely when θ is known with good precision. In this condition all optimal tests should give the best results. In fact, the power of weakly centred tests ($T_{O(1)}^{wc}$ and $T_{O(2)}^{wc}$) is impressive, being around 100% for a large part of the parameter space and decreasing for large migration rates (Figure 3A) and long times (Figure 3B) as every other test, because the frequency spectrum for these cases becomes very similar to the standard spectrum. So, weakly centred tests show a very good theoretical performance, counterbalanced by their lack of robustness. The power of $T_{O(1)}^{wc}$ and $T_{O(2)}^{wc}$ are almost identical, therefore the contribution of the quadratic part to $T_{O(2)}^{wc}$ is probably not relevant.

On the other hand, strongly centred optimal tests are more powerful than Tajima’s *D* but less powerful than weakly centred tests, as expected. However, there is a sensible difference in power between $T_{O(1)}^{sc}$ and $T_{O(2)}^{sc}$: in the range of parameters where the power of weakly centred tests is around 100%, both strongly centred tests show a good performance not so far from the weakly centred ones, while in the less favourable range the quadratic test $T_{O(2)}^{sc}$, while performing worse than the weakly centred tests, has a power that is 20% higher than the linear test $T_{O(1)}^{sc}$. Taking into account the robustness of the tests, these simulations show that optimal tests like $T_{O(2)}^{sc}$ could be an interesting alternative to the usual linear tests.

## 4. Discussion

In this paper we have presented a systematic analysis of neutrality tests based on the site frequency spectrum. This study is intended to extend and complete the recent works in [8,10] by extending the study of the linear neutrality tests presented by Achaz. The properties of linear neutrality tests and optimal tests have been studied using this framework. A new class of “tunable” optimal tests that include usual optimal tests as a special case have been proposed, using a generalisation of the optimisation approach for linear tests.

The aim of the paper is to give mathematical guidelines to build new and more effective tests to detect deviations from neutrality. The proposed guidelines are the scaling relation (8) and the optimality condition based on the maximisation of $E(T_{\Omega })$. Both these guidelines are thoroughly explained and discussed.

One of the strengths of optimal neutrality tests (initially presented in [10]) is the fact that their weights scale automatically with the aim to avoid a dependence with the sample size, enabling interpretation of the results of the test in a sample-size-independent way. For other tests, different scaling strategies were analysed and evaluated, discussing the suitability of weighting methods and the scaling of existing neutrality tests.

The different neutral tests studied and developed in this paper have different features, which make them suitable for different purposes. For example, a general neutrality test where the weights are scaled to obtain interpretable results may be sufficient, with minimum effort, to reject the null model and to interpret results, but the statistical power when faced with a specific alternative hypothesis can be low. Instead, optimal tests become a better approach if the alternative hypothesis is clearly formulated and the data is not clearly from the null hypothesis. In respect to the differences between linear and nonlinear optimal test, while nonlinear optimal tests have been shown to be more powerful than linear ones (and weakly centred tests more powerful than strongly centred ones), power is not the only important issue: robustness must also be taken into consideration.

In fact, there are three important remarks on the relative robustness of these tests. The first one is that, as already discussed, centredness of weakly centred tests is not robust with respect to a biased estimate of θ, therefore these tests should be preferred to strongly centred tests only in situations where the value of θ is well known or a good estimate is available.

The second remark is that neither the weights nor the results of linear optimal tests do depend on the value of θ and on the number of segregating sites *S*, while the weights of nonlinear optimal tests have an explicit dependence on θ and their results depend not only on the spectrum but also on *S*; therefore, the interpretation of the results of these tests is more complicated. However, this is not necessarily true for homogeneous tests of any degree, like the quadratic $G_{\xi }$ test by Fu. An interesting development of this work could be a study of homogeneous tests of a given degree *k* satisfying the optimality condition, which can be easily obtained from Equation (50) by restricting all ordered sequences of indices $\tilde{I},\tilde{J},\tilde{K},\tilde{L}$ to contain precisely *k* indices (along with some “traceless” condition, in case). These homogeneous optimal tests (or at least some subclass of them) should depend weakly on *S*.

The third remark is that linear optimal tests have two interesting properties that are not shared by other tests: they depend only on the deviations from the null spectrum and they have an easy interpretation in terms of these deviations, that is, they are positive if the observed deviations are similar to the expected ones and negative if the observed deviations are opposite to the expected ones. These features give an important advantage to linear optimal tests.

A fourth remark is that “tunable” optimal tests can be built to achieve weights that correspond to maximum power to reject the null hypothesis for a given alternative scenario but uncertain power calculations (e.g., when the variance of the alternative scenario is not known). This new class of neutrality tests is highly flexible and only depends on a single parameter and the mean alternative spectrum. Nevertheless, since the test can be used even when the alternative scenario is not fully defined, there may be situations where optimising its power can result in the interpretability or robustness of the test being compromised.

Tests based on the frequency spectrum of polymorphic sites are fast, being based on simple matrix multiplications, and can therefore be applied to genome-wide data. Moreover, they can be used as summary statistics for Approximate Bayesian Computation or other statistical approaches to the analysis of sequence data. While linear tests are often used in this way, the nonlinear tests presented in this paper contain more information (related to the covariances and higher moments of the frequency spectrum) that could increase the power of these analyses.

## Data Availability

No data was used in this paper.

## Appendix A. Technical Results

### Appendix A.1. Transformations of Weights and Invariance of Tests

We report some theorems on the invariance of the tests under affine transformations. These results can be easily proved and are implicitly used throughout this paper.

**Theorem A1.** 
*A test of the form (3) does not change its value if all the weights $\Omega_{i}$ are rescaled by a common factor λ>0, that is:*

(A1)
$$\Omega_{i}⟶\lambda \Omega_{i}\;\Rightarrow \;T_{\Omega }⟶\mathrm{sign}\left(\lambda \right)T_{\Omega }$$

Note that the invariance of the tests mean that these transformations define equivalence classes of weights, i.e., sets of different weights that actually correspond to the same test. In particular, this theorem implies that the space of possible tests, in terms of the weights $\Omega_{i}$, is not homeomorphic to $R^{n-2}$ (which would be the subspace of weights in $R^{n-1}$ that satisfy the linear condition (4)) but to its quotient with respect to the invariance (multiplicative) group $R^{+}$, that is, the (n−3)-dimensional sphere $S^{n-3}=R^{n-2}/R^{+}$.

**Theorem A2.** 
*A test of the form (7) does not change its value under an affine transformation of parameters $\left(\lambda ,\rho_{i}\right)$ on the weights $\omega_{i}$, $\omega_{i}^{\prime }$ with a common rescaling factor λ>0, that is:*

(A2)
$$\omega_{i}⟶\lambda \omega_{i}+\rho_{i}\;,\;\omega_{i}^{\prime }⟶\lambda \omega_{i}^{\prime }+\rho_{i}\;\Rightarrow \;T_{\Omega }⟶\mathrm{sign}\left(\lambda \right)T_{\Omega }$$

*However, the estimators (5) are unbiased only if the rescaling factor satisfies the condition $\lambda =1-\sum_{i=1}^{n-1}\rho_{i}$.*

### Appendix A.2. Generalised D′ Tests for Multilocus Analysis

The statistic $D^{\prime }$[21], which is defined as the ratio of Tajima’s *D* versus its minimum value (given a fixed number of segregating sites), has been used in the literature for multilocus analyses [21,22,23], arguing that the value of Tajima’s *D* is affected by the length, the sample size, and the number of segregating sites of each studied locus and therefore the values of each locus are not directly comparable.

The contribution of each locus to the heterogeneity is hardly known. Tajima’s *D* is robust to differences in the level of variability (the variance is approximately equal to one) and also quite robust against differences in sample size (as will be shown in the next part), although the quantitative values of Tajima’s *D* for each condition are someway different and therefore the comparison between values is not simple. The proposal of Schaeffer is to use the test:

(A3) $$D^{\prime }=\frac{D}{\min{\left(D\right)}_{S=S_{obs}}}$$

as a (re)normalised version of Tajima’s *D*. $S_{obs}$ is the observed number of segregating sites in the sample. This proposal can be generalised for all the tests of the form (3) as follows:

(A4) $$T_{\Omega }^{\prime }=\frac{T_{\Omega }}{\min{\left(T_{\Omega }\right)}_{S=S_{obs}}}=\frac{\sum_{i=1}^{n-1}i\Omega_{i}\xi_{i}}{\min\left(j\Omega_{j}\right)S_{obs}}$$

This appears to be the natural generalisation of $D^{\prime }$ to general linear tests. For the optimal tests, there are other possibilities.

### Appendix A.3. Tests Based on the Folded SFS

If an outgroup is not available, then the test should be based on the folded spectrum and has the general form:

(A5) $$\begin{array}{cc} T_{\Omega }^{*}= & \frac{{\hat{\theta }}_{\omega }^{*}-{\hat{\theta }}_{\omega^{\prime }}^{*}}{\sqrt{\mathrm{Var}({\hat{\theta }}_{\omega }^{*}-{\hat{\theta }}_{\omega^{\prime }}^{*})}}=\frac{\sum_{i=1}^{\left\lfloor n/2\right\rfloor }i\left(n-i\right)\left(1+\delta_{n,2i}\right)\left(\omega_{i}^{*}-\omega_{i}^{*\prime }\right)\eta_{i}}{\sqrt{\mathrm{Var}\left(\sum_{j=1}^{\left\lfloor n/2\right\rfloor }j\left(n-j\right)\left(1+\delta_{n,2j}\right)\left(\omega_{j}^{*}-\omega_{j}^{*\prime }\right)\eta_{j}\right)}}=\\ = & \frac{\sum_{i=1}^{\left\lfloor n/2\right\rfloor }i\left(n-i\right)\left(1+\delta_{n,2i}\right)\Omega_{i}^{*}\eta_{i}}{\sqrt{\mathrm{Var}\left(\sum_{j=1}^{\left\lfloor n/2\right\rfloor }j\left(n-j\right)\left(1+\delta_{n,2j}\right)\Omega_{j}^{*}\eta_{j}\right)}} \end{array}$$

where the weights $\Omega_{i}^{*}=\omega_{i}^{*}-\omega_{i}^{*\prime }$ satisfy the conditions:

(A6) $$\sum_{i=1}^{\left\lfloor n/2\right\rfloor }\omega_{i}^{*}=1\;,\;\sum_{i=1}^{\left\lfloor n/2\right\rfloor }\omega_{i}^{*\prime }=1\;\Rightarrow \;\sum_{i=1}^{\left\lfloor n/2\right\rfloor }\Omega_{i}^{*}=0$$

and:

(A7) $${\hat{\theta }}_{\omega }^{*}=\frac{1}{L}\sum_{i=1}^{\left\lfloor n/2\right\rfloor }\frac{i\left(n-i\right)\left(1+\delta_{n,2i}\right)}{n}\omega_{i}^{*}\eta_{i}\;,\;{\hat{\theta }}_{\omega^{\prime }}^{*}=\frac{1}{L}\sum_{i=1}^{\left\lfloor n/2\right\rfloor }\frac{i\left(n-i\right)\left(1+\delta_{n,2i}\right)}{n}\omega_{i}^{*\prime }\eta_{i}$$

are unbiased estimators of θ.

### Appendix A.4. Scaling of Weights in Tests without an Outgroup

The above arguments can be repeated in a straightforward way for the tests $T_{\Omega }^{*}$ based on the folded spectrum $\eta_{i}$. The only relevant difference is that the frequency *f* of the minor allele in the population is always less than 50%, that is, f∈(0,1/2]. For consistency with the unfolded case, the weight $\eta_{n/2}$ is reduced by a factor 2. Moreover, the additional parameters related to the weights of singletons cannot distinguish between ancestral and derived alleles and therefore reduce to $\Omega_{s}^{*}$, $\omega_{s}^{*}$, $\omega_{s}^{*\prime }$. These parameters, together with the functions $\Omega^{*}\left(f\right)$, $\omega^{*}\left(f\right)$, and $\omega^{*\prime }\left(f\right)$, should satisfy the conditions:

(A8) $$\Omega_{s}^{*}+\int_{0}^{1/2}df\;\Omega^{*}\left(f\right)=0\;,\;\omega_{s}^{*}+\int_{0}^{1/2}df\;\omega^{*}\left(f\right)=\omega_{s}^{*\prime }+\int_{0}^{1/2}df\;\omega^{*\prime }\left(f\right)=1$$

The formulae that determine the scaling of the weights are:

(A9) $$\Omega_{i}^{*(n)}=\frac{1}{1+\delta_{n,2i}}\Omega^{*}\left(\frac{i}{n}\right)+\Omega_{s}^{*}\delta_{i,1}-\frac{1}{\left\lfloor n/2\right\rfloor }\left(\Omega_{s}^{*}+\sum_{j=1}^{\left\lfloor n/2\right\rfloor }\frac{1}{1+\delta_{n,2j}}\Omega^{*}\left(\frac{j}{n}\right)\right)$$

(A10) $$\Omega_{i}^{*(n)}=\frac{\omega_{s}^{*}\delta_{i,1}+\omega^{*}\left(\frac{i}{n}\right)/\left(1+\delta_{n,2i}\right)}{\omega_{s}^{*}+\sum_{j=1}^{\left\lfloor n/2\right\rfloor }\omega^{*}\left(\frac{j}{n}\right)/\left(1+\delta_{n,2j}\right)}-\frac{\omega_{s}^{*\prime }\delta_{i,1}+\omega^{*\prime }\left(\frac{i}{n}\right)/\left(1+\delta_{n,2i}\right)}{\omega_{s}^{*\prime }+\sum_{j=1}^{\left\lfloor n/2\right\rfloor }\omega^{*\prime }\left(\frac{j}{n}\right)/\left(1+\delta_{n,2j}\right)}$$

The weights of the folded versions of Tajima’s *D* and Fu and Li’s $F^{*}$ test follow this scaling. The nonzero weight functions are $\omega^{*}\left(f\right)=1$, $\omega^{*\prime }\left(f\right)=1/\left(\log\left(N\right)f\left(1-f\right)\right)$ for Tajima’s *D* and $\omega_{s}^{*}=1$, $\omega^{*\prime }\left(f\right)=1/\left(\log\left(N\right)f\left(1-f\right)\right)$ for the test of Fu and Li.

### Appendix A.5. Scaling of Optimal Tests

Optimal tests have weights proportional to the expected allele distribution $\omega_{i}={\bar{\xi }}_{i}/\sum_{j=1}^{n-1}{\bar{\xi }}_{j}$ and to the null allele distribution $\omega_{i}^{\prime }=\xi_{i}^{0}/\sum_{j=1}^{n-1}\xi_{j}^{0}$. Therefore, the weights of an optimal test follow the same scaling with sample size as the allele distributions. Denoting by $\bar{\xi }\left(f\right)$ and $\xi^{0}\left(f\right)$ the spectra of expected and null allele frequencies in the whole population, the spectra for the sample are obtained by binomial sampling:

(A11) ξi=∫1/Ne1dfPbin(i;n,f)ξ(f)Pbin(i;n,f)=nifi(1−f)n−i

from the spectra $\xi \left(f\right)=\bar{\xi }\left(f\right)$ and $\xi \left(f\right)=\xi^{0}\left(f\right)$, respectively. Therefore the scaling of the allele distributions is:

(A12) ξ¯i∑j=1n−1ξ¯j=∫1/Ne1dfnifi(1−f)n−iξ¯(f)∫1/Ne1df(1−fn−(1−f)n)ξ¯(f)

(A13) ξi0∑j=1n−1ξj0=∫1/Ne1dfnifi(1−f)n−iξ0(f)∫1/Ne1df(1−fn−(1−f)n)ξ0(f)

which is precisely the scaling (26) and (27) with weight functions $\omega \left(f\right)\propto \bar{\xi }\left(f\right)$ and $\omega^{\prime }\left(f\right)\propto \xi^{0}\left(f\right)$. This scaling does not correspond to the scaling (8) suggested in this paper, but it takes into account the sampling process in a straightforward way, being based on the expected and null allele distributions for the sample and therefore immediately related to the binomial sampling of alleles from the population.

Note that these weights actually follow the scaling (8) for large *n*, with the same weight functions $\omega \left(f\right)\propto \bar{\xi }\left(f\right)$ and $\omega^{\prime }\left(f\right)\propto \xi^{0}\left(f\right)$. This agrees with the fact that when the sample size is large enough, the variance of the sampling process can be safely ignored and all reasonable choices of scaling are equivalent to our proposal (8).

### Appendix A.6. Generalising D′ for Optimal Test

The $D^{\prime }$ statistics of [21] can be generalised for optimal tests as it was done for general linear tests. In particular, for a fixed number of segregating sites $S_{obs}$, the generalisation for optimal tests in the approximation of unlinked sites and θ≪1 is:

(A14) $$T_{O}^{\prime }=\frac{\sum_{i=1}^{n-1}\Omega_{i}\xi_{i}/\xi_{i}^{0}}{\min_{j}\left(\Omega_{j}/\xi_{j}^{0}\right)S_{obs}}=\frac{\sum_{i=1}^{n-1}\left(\xi_{i}/S_{obs}\right)\left({\bar{\xi }}_{i}/\sum_{j=1}^{n-1}{\bar{\xi }}_{j}\right)/\left(\xi_{i}^{0}/\sum_{j=1}^{n-1}\xi_{j}^{0}\right)-1}{\min_{k}\left(\left({\bar{\xi }}_{k}/\sum_{l=1}^{n-1}{\bar{\xi }}_{l}\right)/\left(\xi_{k}^{0}/\sum_{l=1}^{n-1}\xi_{l}^{0}\right)\right)-1}$$

which has the interesting property of depending only on the allele frequency distributions.

However, in the case of optimal tests there is another possibility, namely to define a ${\bar{D}}^{\prime }$ test as the ratio of the optimal test and of its average minimum, assuming that the spectrum corresponds to the average spectrum of the actual scenario. This would correspond to the form:

(A15) $${\bar{T}}_{O}^{\prime }=-\frac{\langle \langle \xi ,\bar{\xi }\rangle \rangle -\langle \langle \xi ,\xi^{0}\rangle \rangle \langle \langle \xi^{0},\bar{\xi }\rangle \rangle /\langle \langle \xi^{0},\xi^{0}\rangle \rangle }{S_{obs}/a_{n}\sqrt{\left(\langle \langle \bar{\xi },\bar{\xi }\rangle \rangle -{\langle \langle \xi^{0},\bar{\xi }\rangle \rangle }^{2}/\langle \langle \xi^{0},\xi^{0}\rangle \rangle \right)\left(\langle \langle \xi ,\xi \rangle \rangle -{\langle \langle \xi^{0},\xi \rangle \rangle }^{2}/\langle \langle \xi^{0},\xi^{0}\rangle \rangle \right)}}$$

which has the interesting property of being symmetric with respect to the actual spectrum ξ and the expected spectrum $\bar{\xi }$.

### Appendix A.7. Moments of the Frequency Spectrum in the Independent Sites Approximation

We consider the limit θ≪1, L→∞ with constant θL. The spectrum $\xi_{i}$ can be written as a sum of spectra for all sites:

(A16) $$\xi_{i}=\sum_{s=1}^{L}\xi_{i}\left(s\right)$$

where each variable $\xi_{i}\left(s\right)$ has a Bernoulli distribution $\xi_{i}\left(s\right)\in \left\{0,1\right\}$ with probabilities $p\left(1\right)=\theta \xi_{i}^{0}$ and $p\left(0\right)=1-\theta \xi_{i}^{0}$ where $\xi_{i}^{0}=1/i$ under the standard neutral model. The expectation value of $\xi_{i}$ is therefore:

(A17) $$E\left(\xi_{i}\right)=\mu_{i}=\sum_{s=1}^{L}E\left(\xi_{i}\left(s\right)\right)=\theta L\xi_{i}^{0}=\frac{\theta L}{i}$$

and similarly $E\left(\xi_{i}\right)={\bar{\mu }}_{i}=\theta L{\bar{\xi }}_{i}$ for a general model with average spectrum ${\bar{\xi }}_{i}$.

In the independent sites approximation, which is equivalent to the infinite recombination limit, the variables $\xi_{i}\left(s\right)$ and $\xi_{i}\left(s^{\prime }\right)$ are i.i.d. random variables for $s\neq s^{\prime }$, and more generally the random variables $\xi_{i}\left(s\right)$ and $\xi_{j}\left(s^{\prime }\right)$ are independent for $s\neq s^{\prime }$. The moments for a single site *s* can be calculated as:

(A18) $$\begin{array}{cc} E(\xi_{i}\left(s\right)\xi_{j}\left(s\right)\xi_{k}\left(s\right)\ldots )= & \sum_{a,b,c\ldots \in \{0,1\}}abc\ldots P\left(\xi_{i}\left(s\right)=a,\xi_{j}\left(s\right)=b,\xi_{k}\left(s\right)=c,\ldots \right)=\\ =P(\xi_{i}\left(s\right)=1,\xi_{j}\left(s\right)= & 1,\xi_{k}\left(s\right)=1,\ldots )=P\left(\xi_{i}\left(s\right)=1\right)\delta_{ij}\delta_{jk}\ldots \end{array}$$

because the sum $\sum_{i=1}^{n-1}\xi_{i}\left(s\right)\in \left\{0,1\right\}$, that is, different allele frequencies for the same site are mutually exclusive. Therefore all these moments are always linear in θ and are nonzero only when all indices are equal:

(A19) $$E\left(\xi_{i}\left(s\right)\xi_{j}\left(s\right)\xi_{k}\left(s\right)\ldots \right)=\theta \xi_{i}^{0}\delta_{ij}\delta_{jk}\ldots =\frac{\theta }{i}\delta_{ij}\delta_{jk}\ldots \;.$$

Then the second moment can be evaluated as:

(A20) $$\begin{array}{cc} E(\xi_{i}\xi_{j})= & \sum_{s,s^{\prime }}E\left(\xi_{i}\left(s\right)\xi_{j}\left(s^{\prime }\right)\right)=\sum_{s}E\left(\xi_{i}\left(s\right)\xi_{j}\left(s\right)\right)+\sum_{s\neq s^{\prime }}E\left(\xi_{i}\left(s\right)\right)E\left(\xi_{j}\left(s^{\prime }\right)\right)=\\ = & L\delta_{ij}\frac{\theta }{i}+L\left(L-1\right)\frac{\theta^{2}}{ij}=\delta_{ij}\mu_{i}+\mu_{i}\mu_{j}-\frac{\mu_{i}\mu_{j}}{L} \end{array}$$

and neglecting subleading orders in θ or $L^{-1}$ like the last term above, we can calculate the third and forth moments that are needed for the calculation of $C^{-1}$.

The final result for the moments of the spectrum $\xi_{i}$ is:

(A21) $$\begin{array}{cc} E\left(\xi_{i}\xi_{j}\right)=\mu_{ij}= & \delta_{ij}\mu_{i}+\mu_{i}\mu_{j} \end{array}$$

(A22) $$\begin{array}{cc} E\left(\xi_{i}\xi_{j}\xi_{k}\right)=\mu_{ijk}= & \delta_{ij}\delta_{jk}\mu_{i}+\left(\delta_{ik}\mu_{i}\mu_{j}+\delta_{jk}\mu_{i}\mu_{j}+\delta_{ij}\mu_{i}\mu_{k}\right)+\mu_{i}\mu_{j}\mu_{k} \end{array}$$

(A23) $$\begin{array}{cc} E\left(\xi_{i}\xi_{j}\xi_{k}\xi_{l}\right)=\mu_{ijkl}= & \delta_{ij}\delta_{jk}\delta_{kl}\mu_{i}+\left(\delta_{ik}\delta_{jl}\mu_{i}\mu_{j}+\delta_{il}\delta_{jk}\mu_{i}\mu_{j}+\delta_{ij}\delta_{kl}\mu_{i}\mu_{k}\right)+\\ + & (\delta_{ij}\delta_{jk}\mu_{i}\mu_{l}+\delta_{ij}\delta_{jl}\mu_{i}\mu_{k}+\delta_{ik}\delta_{kl}\mu_{i}\mu_{j}+\delta_{jk}\delta_{kl}\mu_{i}\mu_{j})+\\ + & (\delta_{il}\mu_{i}\mu_{j}\mu_{k}+\delta_{jl}\mu_{i}\mu_{j}\mu_{k}+\delta_{ik}\mu_{i}\mu_{j}\mu_{l}+\delta_{jk}\mu_{i}\mu_{j}\mu_{l}+\\ + & \delta_{ij}\mu_{i}\mu_{k}\mu_{l}+\delta_{kl}\mu_{i}\mu_{j}\mu_{k})+\mu_{i}\mu_{j}\mu_{k}\mu_{l} \end{array}$$

All the results above can be applied to a general model simply by substituting $\mu_{i}$ with ${\bar{\mu }}_{i}$. Moreover, they can be applied to the folded spectrum by taking $\mu_{i}=\theta Ln/i\left(n-i\right)\left(1+\delta_{n,2i}\right)$ for the standard neutral model or ${\bar{\mu }}_{i}=\theta L({\bar{\xi }}_{i}+{\bar{\xi }}_{n-i})/\left(1+\delta_{n,2i}\right)$ for general models.

We define some quantities in order to simplify the expressions for the weights:

(A24) $$\begin{array}{cccccc} \Sigma_{\mu } & =\sum_{i=1}^{n-1}\mu_{i}\;, & \;\Sigma_{\bar{\mu }} & =\sum_{i=1}^{n-1}{\bar{\mu }}_{i}\;, & \;\Sigma_{q} & =\sum_{i=1}^{n-1}\frac{{\bar{\mu }}_{i}^{2}}{\mu_{i}} \end{array}$$

If the spectrum is folded, all the sums in the above expressions run from 1 to ⌊n/2⌋.

The covariance matrix $C_{\tilde{I},\tilde{J}}$ is:

(A25) $$\begin{array}{cc} C_{i,j} & =\mu_{ij}-\mu_{i}\mu_{j}=\delta_{ij}\mu_{i} \end{array}$$

(A26) $$\begin{array}{cc} C_{ij,k} & =\mu_{ijk}-\mu_{ij}\mu_{k}=\delta_{ij}\delta_{jk}\mu_{i}+\left(\delta_{ik}\mu_{i}\mu_{j}+\delta_{jk}\mu_{i}\mu_{j}\right) \end{array}$$

(A27) $$\begin{array}{cc} C_{ij,kl} & =\mu_{ijkl}-\mu_{ij}\mu_{kl}=\delta_{ij}\delta_{jk}\delta_{kl}\mu_{i}+\left(\delta_{ik}\delta_{jl}\mu_{i}\mu_{j}+\delta_{il}\delta_{jk}\mu_{i}\mu_{j}\right)+\\ \; & +(\delta_{ij}\delta_{jk}\mu_{i}\mu_{l}+\delta_{ij}\delta_{jl}\mu_{i}\mu_{k}+\delta_{ik}\delta_{kl}\mu_{i}\mu_{j}+\delta_{jk}\delta_{kl}\mu_{i}\mu_{j})+\\ \; & +(\delta_{il}\mu_{i}\mu_{j}\mu_{k}+\delta_{jl}\mu_{i}\mu_{j}\mu_{k}+\delta_{ik}\mu_{i}\mu_{j}\mu_{l}+\delta_{jk}\mu_{i}\mu_{j}\mu_{l}) \end{array}$$

with the elements $C_{ij,k}$ and $C_{ij,kl}$ that should be considered only for i≤j, k≤l. It can be verified that the inverse matrix $C_{\tilde{I},\tilde{J}}^{-1}$ has the form:

(A28) $$\begin{array}{cc} C_{i,j}^{-1}= & 1+\delta_{ij}\frac{2\mu_{i}\left(\Sigma_{\mu }+3\right)+1}{2\mu_{i}^{2}} \end{array}$$

(A29) $$\begin{array}{cc} C_{ij,k}^{-1}= & \delta_{ij}\delta_{jk}\frac{2\mu_{i}-1}{2\mu_{i}^{2}}-\left(\delta_{ik}+\delta_{jk}\right)\frac{1}{\mu_{k}} \end{array}$$

(A30) $$\begin{array}{cc} C_{ij,kl}^{-1}= & \left(\delta_{ik}\delta_{jl}+\delta_{il}\delta_{jk}\right)\frac{1}{\mu_{i}\mu_{j}}-\frac{3}{2}\delta_{ij}\delta_{ik}\delta_{jl}\frac{1}{\mu_{i}^{2}} \end{array}$$

The formulae for the weakly centred quadratic test (66)–(69) can be obtained from these formulae and the definition (63). The corresponding variance in the denominator (45) is:

(A31) $$\begin{array}{ccc} \mathrm{Var}\left(\sum_{I}\Gamma_{I}^{\left(n_{I}\right)}{\left(\xi \ldots \xi \right)}_{I}\right) & = & 2{\left(\Sigma_{\bar{\mu }}-\Sigma_{q}/2\right)}^{2}+\Sigma_{\mu }\left(\Sigma_{\mu }/2+1-2\Sigma_{\bar{\mu }}+\Sigma_{q}\right)\\ & & +\Sigma_{q}-2\Sigma_{\bar{\mu }} \end{array}$$

The matrix M is the inverse of the matrix $M_{rl}^{-1}=\sum_{\tilde{I},\tilde{L}}\mu_{\tilde{I}}^{\left(r\right)}C_{\tilde{I}\tilde{L}}^{-1}\mu_{\tilde{L}}^{\left(l\right)}$, which can be easily calculated from the above equations as:

(A32) $$\begin{array}{cc} M_{11}^{-1} & =2\Sigma_{\mu }^{2}+\Sigma_{\mu } \end{array}$$

(A33) $$\begin{array}{cc} M_{12}^{-1} & =-\Sigma_{\mu }^{2} \end{array}$$

(A34) $$\begin{array}{cc} M_{22}^{-1} & =\Sigma_{\mu }^{2}/2 \end{array}$$

and the matrix M is

(A35) $$M=\left(\begin{array}{cc} 1/\Sigma_{\mu } & 2/\Sigma_{\mu }\\ 2/\Sigma_{\mu } & 4/\Sigma_{\mu }+2/\Sigma_{\mu }^{2} \end{array}\right)$$

The formulae (54)–(56) can be obtained from the formula (A35) and from the following results:

(A36) $$\begin{array}{cc} \sum_{\tilde{I}}C_{i,\tilde{I}}^{-1}{\bar{\mu }}_{\tilde{I}} & =\Sigma_{\bar{\mu }}+\left(\Sigma_{\mu }+2-\Sigma_{\bar{\mu }}\right)\frac{{\bar{\mu }}_{i}}{\mu_{i}}-\frac{1}{2}{\left(\frac{{\bar{\mu }}_{i}}{\mu_{i}}\right)}^{2} \end{array}$$

(A37) $$\begin{array}{cc} \sum_{\tilde{I}}C_{ii,\tilde{I}}^{-1}{\bar{\mu }}_{\tilde{I}} & =-\frac{{\bar{\mu }}_{i}}{\mu_{i}}+\frac{1}{2}{\left(\frac{{\bar{\mu }}_{i}}{\mu_{i}}\right)}^{2} \end{array}$$

(A38) $$\begin{array}{cc} \sum_{\tilde{I}}C_{ij,\tilde{I}}^{-1}{\bar{\mu }}_{\tilde{I}} & =-\frac{{\bar{\mu }}_{i}}{\mu_{i}}-\frac{{\bar{\mu }}_{j}}{\mu_{j}}+\frac{{\bar{\mu }}_{i}{\bar{\mu }}_{j}}{\mu_{i}\mu_{j}} \end{array}$$

(A39) $$\begin{array}{cccc} \sum_{\tilde{I},\tilde{J}}\mu_{\tilde{I}}^{\left(1\right)}C_{\tilde{I}\tilde{J}}^{-1}{\bar{\mu }}_{\tilde{J}} & =\Sigma_{\bar{\mu }}\left(2\Sigma_{\mu }-\Sigma_{\bar{\mu }}+1\right)\; & \sum_{\tilde{I},\tilde{J}}\mu_{\tilde{I}}^{\left(2\right)}C_{\tilde{I}\tilde{J}}^{-1}{\bar{\mu }}_{\tilde{J}} & =\Sigma_{\bar{\mu }}^{2}/2-\Sigma_{\mu }\Sigma_{\bar{\mu }} \end{array}$$

(A40) $$\begin{array}{cccc} \sum_{\tilde{I}}C_{i,\tilde{I}}^{-1}\mu_{\tilde{I}}^{\left(1\right)} & =2\Sigma_{\mu }+2\; & \sum_{\tilde{I}}C_{i,\tilde{I}}^{-1}\mu_{\tilde{I}}^{\left(2\right)} & =-\Sigma_{\mu }-1/2 \end{array}$$

(A41) $$\begin{array}{cccc} \sum_{\tilde{I}}C_{ii,\tilde{I}}^{-1}\mu_{\tilde{I}}^{\left(1\right)} & =-1\; & \sum_{\tilde{I}}C_{ii,\tilde{I}}^{-1}\mu_{\tilde{I}}^{\left(2\right)} & =1/2 \end{array}$$

(A42) $$\begin{array}{cccc} \sum_{\tilde{I}}C_{ij,\tilde{I}}^{-1}\mu_{\tilde{I}}^{\left(1\right)} & =-2\; & \sum_{\tilde{I}}C_{ij,\tilde{I}}^{-1}\mu_{\tilde{I}}^{\left(2\right)} & =1 \end{array}$$

## Appendix B. Proofs

### Derivation of General Optimisation for Tunable Linear Tests

We consider the maximisation of M(E,V) with the conditions $E_{0}=0$, $V_{0}=1$. We assume that the function M(E,V) is monotonic in both variables.

The existence of an absolute maximum (and minimum) is ensured by Weierstrass theorem applied to the closed manifold determined by the conditions $E_{0}=0$, $V_{0}=1$ [10].

Local maxima can be found by the Lagrange multipliers method, i.e., by the extremum conditions for $M\left(E,V\right)-\lambda_{1}E_{0}-\lambda_{2}\left(V_{0}-1\right)$ with respect to $\Omega_{i}$, $\lambda_{1}$ and $\lambda_{2}$. We obtain the extremum equation:

(A43) $$\Omega_{i}=-\frac{\theta L}{2}\frac{\partial M(E,V)/\partial E}{\partial M(E,V)/\partial V}\cdot \xi_{i}^{0}\sum_{j=1}^{n-1}{\left(\bar{c}+{\tilde{\lambda }}_{2}c\right)}_{ij}^{-1}\left({\bar{\xi }}_{j}-{\tilde{\lambda }}_{1}\xi_{j}^{0}\right)$$

with ${\tilde{\lambda }}_{1}$, ${\tilde{\lambda }}_{2}$ determined by the constraint equations. Solving for ${\tilde{\lambda }}_{1}$, we obtain:

(A44) $$\Omega_{i}=-\frac{\theta L}{2}\frac{\partial M(E,V)/\partial E}{\partial M(E,V)/\partial V}\cdot \xi_{i}^{0}\sum_{j=1}^{n-1}{\left(\bar{c}+{\tilde{\lambda }}_{2}c\right)}_{ij}^{-1}\left[{\bar{\xi }}_{j}-\xi_{j}^{0}\frac{\sum_{l,m=1}^{n-1}\xi_{l}^{0}{\left(\bar{c}+{\tilde{\lambda }}_{2}c\right)}_{lm}^{-1}{\bar{\xi }}_{m}}{\sum_{l,m=1}^{n-1}\xi_{l}^{0}{\left(\bar{c}+{\tilde{\lambda }}_{2}c\right)}_{lm}^{-1}\xi_{m}^{0}}\right]$$

with ${\tilde{\lambda }}_{2}$ determined by the condition $\sum_{i,j=1}^{n-1}\Omega_{i}\Omega_{j}c_{ij}/\xi_{i}^{0}\xi_{j}^{0}=1$.

We approximate the variances with their values for unlinked sites:

(A45) $$c_{ij}≃\delta_{ij}\theta L\xi_{i}^{0}\;,\;{\bar{c}}_{ij}≃\delta_{ij}\theta L{\bar{\xi }}_{i}$$

and we obtain the weights:

(A46) $$\Omega_{i}=\xi_{i}^{0}\left(1-\frac{\sum_{j=1}^{n-1}\xi_{j}^{0}}{\left(\lambda {\bar{\xi }}_{i}/\xi_{i}^{0}+1\right)\sum_{j=1}^{n-1}\frac{\xi_{j}^{0}}{\lambda {\bar{\xi }}_{j}/\xi_{j}^{0}+1}}\right)$$

where the remaining parameter λ is fixed by the condition:

(A47) $$\frac{\theta^{3}L^{3}}{4}\sum_{i=1}^{n-1}\Omega_{i}^{2}/\xi_{i}^{0}={\left(\frac{\partial M(E,V)/\partial V}{\partial M(E,V)/\partial E}\right)}^{2}$$

This equation fixes all values of λ corresponding to the local maxima. To obtain the absolute maximum, one has to choose the solution λ that actually maximises M(E,V).

In practice, for the standard neutral model, it is sufficient to use the usual form of the test:

(A48) $$T=\frac{\sum_{i=1}^{n-1}i\Omega_{i}\xi_{i}}{\sqrt{\mathrm{Var}(\sum_{i=1}^{n-1}i\Omega_{i}\xi_{i})}}$$

with the (unnormalised) weights:

(A49) $$\Omega_{i}=\frac{1}{i}\left(1-\frac{a_{n}}{\left(\lambda i{\bar{\xi }}_{i}+1\right)\sum_{j=1}^{n-1}\frac{1}{j(\lambda j{\bar{\xi }}_{j}+1)}}\right)$$

with λ determined numerically either by the condition:

(A50) $$\frac{\theta^{3}L^{3}}{4}\sum_{i=1}^{n-1}i\Omega_{i}^{2}={\left(\frac{\partial M(E,V)/\partial V}{\partial M(E,V)/\partial E}\right)}^{2}$$

or more easily, looking directly for the value of λ that maximises numerically M(E,V).

We discuss explicitly two important limit cases:

- ∂M/∂V=0: this case reduces to the usual optimal tests. It corresponds to the limit $\lambda \to 0^{+}$ in the above equation, which gives a unique solution:
  (A51) $$\Omega_{i}=\lambda \left({\bar{\xi }}_{i}-\frac{1}{i}\frac{\sum_{j=1}^{n-1}{\bar{\xi }}_{j}}{a_{n}}\right)$$
  where the λ factor is irrelevant.
- ∂M/∂E=0: maximisation or minimisation of variance. In this case the values of λ are determined by the condition $\sum_{j=1}^{n-1}\frac{1}{j(\lambda j{\bar{\xi }}_{j}+1)}=0$, which is equivalent to $a_{n}=\sum_{j=1}^{n-1}\frac{1}{j+1/\lambda {\bar{\xi }}_{j}}$. The r.h.s. is monotonically increasing and diverges for the n−1 values $\lambda =-1/i{\bar{\xi }}_{i}$, i=1…n−1. Monotonicity and the Weierstrass theorem ensure that there are precisely n−2 solutions for this equation. If we order the values of $i{\bar{\xi }}_{i}$ from the lowest $i_{1}{\bar{\xi }}_{i_{1}}$ to the highest $i_{n-1}{\bar{\xi }}_{i_{n-1}}$, the value of λ that maximises V is the only solution that lies in the interval $-1/i_{1}{\bar{\xi }}_{i_{1}}<\lambda <-1/i_{2}{\bar{\xi }}_{i_{2}}$ and the value that minimises V is the only solution in $-1/i_{n-2}{\bar{\xi }}_{i_{n-2}}<\lambda <-1/i_{n-1}{\bar{\xi }}_{i_{n-1}}$.

**Proof of Theorem 1.** Choose a vector $\Delta_{i}$ in $R^{n-1}$ that is orthogonal both to $i\Omega_{i}$ and to *i*, that is, such that $\sum_{i}i\Delta_{i}\Omega_{i}=0$ and $\sum_{i}i\Omega_{i}=0$. Since $\alpha /ia_{n}+\left(1-\alpha \right)\Delta_{i}$ is a set of continuous functions of α, its minimum is also continuous in α. Moreover, the minimum is clearly positive if α=1, while it is negative by construction if α=0. The theorem follows from the intermediate value theorem. □

**Proof of Theorem 2.** The proof is similar to the previous one. Choose a function Δ(f) in $C^{\infty }\left(0,1\right)$ to satisfy both $\int_{1/N}^{1}df\;f\Omega \left(f\right)\Delta \left(f\right)=0$ and $\int_{1/N}^{1}df\;f\Delta \left(f\right)=0$. (Since these conditions correspond just to two independent functionals of Δ(f) and $C^{\infty }\left(0,1\right)$ is an infinite-dimensional linear space, the existence of such a function is guaranteed). Since α/flog(N)+(1−α)Δ(f) is a continuous functions of α and its infimum is not ±∞, its infimum is also continuous in α. Moreover, the infimum is clearly positive if α=1, while it is negative by construction if α=0. The theorem follows from the intermediate value theorem. □

**Proof of Theorem 3.** The vectors $\Omega_{i}$ are a basis of the subspace $R^{n-2}\subset R^{n-1}$ defined by the condition $\sum_{i}\Omega_{i}=0$, that is, the space of vectors orthogonal to the vector whose components are $v_{i}=1$. Therefore the only vectors $i\Delta_{i}$ that are orthogonal to all the vectors in this basis are precisely of the form $i\Delta_{i}\propto v_{i}$, that is, $\Delta_{i}=const/i$. □

The theorems on the form of optimal tests can be easily proved from a general lemma.

**Lemma A1.** 
*Consider a function $f:R^{M}\setminus \left\{0\right\}\to R$ of the form:*

(A52)
$$f\left(\vec{v}\right)=\frac{\vec{v}\cdot \vec{w}}{\sqrt{\vec{v}\cdot Q\vec{v}}}$$

*where $\vec{w}\in R^{M}$ and Q is a M×M symmetric positive matrix, and a K×M matrix R with K<M and maximum rank. The extrema of the function f restricted to the subspace $R\vec{v}=0$ are given by:*

(A53)
$${\vec{v}}_{\alpha }=\alpha \left(Q^{-1}\vec{w}-Q^{-1}R^{t}{\left(RQ^{-1}R^{t}\right)}^{-1}RQ^{-1}\vec{w}\right)$$

*The extrema with α>0 are maxima and the extrema with α<0 are minima of the function f. These extrema satisfy the identity:*

(A54)
$${\vec{v}}_{\alpha }\cdot Q{\vec{v}}_{\alpha }=\alpha {\vec{v}}_{\alpha }\cdot \vec{w}$$

**Proof.** The existence of maxima and minima can be proved by the Weierstrass extreme value theorem. In fact *f* is continuous and invariant under a homothety with centre in the origin of $R^{M}$ and positive scale factor, therefore the codomain of the function on the linear subspace defined by $R\vec{v}=0$ is the same as the codomain of its restriction to the submanifold of unit vectors $|\vec{v}|=1$, which is a compact space. The restriction of *f* is also continuous and the conclusion follows. To determine the extrema, the method of Lagrange multipliers states that it is sufficient to extremise the function:

(A55) $$F\left(\vec{v},\vec{\lambda }\right)=\frac{\vec{v}\cdot \vec{w}}{\sqrt{\vec{v}\cdot Q\vec{v}}}+\vec{\lambda }\cdot R\vec{v}$$

and since there are no boundaries, this is equivalent to the solution of the equations:

(A56) $$\begin{array}{cc} 0={\vec{\nabla }}_{v}f= & \frac{\vec{w}}{\sqrt{\vec{v}\cdot Q\vec{v}}}-\frac{\vec{v}\cdot \vec{w}}{{\left(\vec{v}\cdot Q\vec{v}\right)}^{3/2}}Q\vec{v}+R^{t}\vec{\lambda } \end{array}$$

(A57) $$\begin{array}{cc} 0={\vec{\nabla }}_{\lambda }f= & R\vec{v} \end{array}$$

The solution satisfies:

(A58) $$\frac{\vec{v}\cdot \vec{w}}{\vec{v}\cdot Q\vec{v}}\vec{v}=Q^{-1}\vec{w}+Q^{-1}R^{t}\vec{\lambda }$$

and multiplying it by *R* and using (A57) we obtain:

(A59) $$\vec{\lambda }=-{\left(RQ^{-1}R^{t}\right)}^{-1}RQ^{-1}\vec{w}+\vec{l}\;,\;R^{t}\vec{l}=0$$

that can be inserted again in Equation (A58) to eliminate $\vec{\lambda }$. The resulting equation in $\vec{v}$ admits only solutions of the form (A53) and by substituting (A53) into it, it can be checked that all values of α≠0 correspond to solutions of (A56) and (A57). The invariance of *f* under a central homothety with positive scale factor implies that the value of the function does not depend on |α|. The function is positive for α>0 and negative for α<0, therefore solutions with α>0 correspond to maxima and solutions with α<0 correspond to minima. The identity (A54) can be proved by substituting the solution (A53). □

**Proof of Theorem 4.** The expected values of the tests (41) have the same functional form as the function *f* of Lemma A1. The correspondence is the following:

(A60) $$\begin{array}{cc} \vec{v}\;\to \; & v_{\tilde{I}}=\sigma \left(\tilde{I}\right)\Omega_{\tilde{I}}^{\left(n_{\tilde{I}}\right)} \end{array}$$

(A61) $$\begin{array}{cc} \vec{w}\;\to \; & w_{\tilde{I}}={\bar{\mu }}_{\tilde{I}} \end{array}$$

(A62) $$\begin{array}{cc} Q\;\to \; & Q_{\tilde{I}\tilde{J}}=\mu_{\tilde{I}\tilde{J}}-\mu_{\tilde{I}}\mu_{\tilde{J}} \end{array}$$

(A63) $$\begin{array}{cc} R\;\to \; & R_{k,\tilde{I}}=\mu_{\tilde{I}}^{\left(k\right)} \end{array}$$

and the positivity of the matrix *Q* is guaranteed by the positivity of the variance for all possible choices of the weights. Application of Lemma A1 with a α=1 gives the result (50). □

**Proof of Theorem 5.** We can immediately solve Equation (58) for γ and substitute it in Equation (57). Then γ is a function of the other weights and the maximisation is unconstrained. It can be seen that also in this case, the expected values of the tests have the same functional form as the function *f* of Lemma A1. The correspondence is the following:

(A64) $$\begin{array}{cc} \vec{v}\;\to \; & v_{\tilde{I}}=\sigma \left(\tilde{I}\right)\Gamma_{\tilde{I}}^{\left(n_{\tilde{I}}\right)} \end{array}$$

(A65) $$\begin{array}{cc} \vec{w}\;\to \; & w_{\tilde{I}}={\bar{\mu }}_{\tilde{I}}-\mu_{\tilde{I}} \end{array}$$

(A66) $$\begin{array}{cc} Q\;\to \; & Q_{\tilde{I}\tilde{J}}=\mu_{\tilde{I}\tilde{J}}-\mu_{\tilde{I}}\mu_{\tilde{J}} \end{array}$$

(A67) $$\begin{array}{cc} R\;\to \; & \mathrm{empty}\;0\times M\;\mathrm{matrix} \end{array}$$

and the positivity of the matrix *Q* is implied by by the positivity of the variance. Then the result (63) follows from Lemma A1 with α=1. □
